# Coordinated actions of NLR-assembled and glutamate receptor–like calcium channels in plant effector-triggered immunity

**DOI:** 10.1073/pnas.2508018122

**Published:** 2025-08-22

**Authors:** Junli Wang, Xinhua Sun, Fei Xiong, Dmitry Lapin, Tak Lee, Sergio Martin-Ramirez, Anna Prakken, Qiaochu Shen, Jaqueline Bautor, Takaki Maekawa, Jane E. Parker

**Affiliations:** ^a^Department of Plant-Microbe Interactions, Max Planck Institute for Plant Breeding Research, Cologne 50829, Germany; ^b^Institute for Plant Sciences, University of Cologne, Cologne, North Rhine-Westphalia 50674, Germany; ^c^Cluster of Excellence on Plant Sciences, Max Planck Institute for Plant Breeding Research, Cologne, North Rhine-Westphalia 50829, Germany

**Keywords:** effector-triggered immunity, N requirement gene 1, glutamate receptor–like, calcium, *Nicotiana benthamiana*

## Abstract

The plant immune system utilizes nucleotide-binding/leucine-rich repeat (NLR) proteins to detect pathogen virulence factors (effectors) inside host cells and transduce recognition to rapid defense. In dicotyledenous plants, pathogen activated Toll-like/interleukin-1 receptor-containing NLRs (TNLs) establish a signaling network of enhanced susceptibility 1 (EDS1)-family dimers with RPW8-type coiled-coil (CC_R_) domain NLRs (RNLs) to stimulate transcriptional reprogramming leading to host cell death and pathogen restriction. Evidence suggests that TNL- and EDS1-activated RNLs function as oligomeric Ca^2+^ permeable ion channels at the plasma membrane. However, the downstream processes for immunity execution are poorly understood. Here, we studied pathogen effector-triggered immunity conferred by *Nicotiana benthamiana* TNL (Roq1) which signals almost exclusively through the EDS1-senescence associated gene101 (SAG101)-N required gene 1 (NRG1) RNL module. We identify a pair of glutamate receptor–like Ca^2+^ ion channels (GLR2.9a and GLR2.9b) which, unlike most other pathogen-induced GLRs, are highly up-regulated by the EDS1-SAG101-NRG1 module in the TNL immune response. We show that oligomeric NRG1 Ca^2+^ channel activity is necessary for *GLR2.9a* and *GLR2.9b* induced expression. Consequently, GLR2.9a and GLR2.9b proteins contribute to *NRG1*-dependent Ca^2+^ accumulation in host cells, and to pathogen resistance and host cell death. We establish that GLR2.9a localizes mainly to the plasma membrane/cytoplasm whereas GLR2.9b accumulates preferentially at the nuclear envelope. The data show that transcriptionally up-regulated canonical Ca^2+^ ion channels GLR2.9a and GLR2.9b are a functional output of the EDS1-SAG101-NRG1 module for TNL-triggered immunity.

## Significance

A major barrier to microbial infection in plants is mediated by large families of intracellular nucleotide-binding/leucine-rich repeat (NLR) immune receptors recognizing pathogen attack. Significant advances have been made in understanding mechanisms of NLR activation and early signaling, with some NLRs functioning as pathogen-induced oligomeric Ca^2+^ permeable ion channels which promote host transcriptional changes and cell death at pathogen infection sites. The next steps in immune response execution remain obscure. Here, we identify and characterize a pair of glutamate receptor–like (GLR) Ca^2+^ ion channels as one important NLR-controlled transcriptional immune output. Our analysis shows that NLR-assembled and canonical Ca^2+^ ion channels cooperate to confer robust resistance against disease.

Plants have evolved sophisticated innate immune systems which deploy cell-surface and intracellular receptors to detect pathogen attack and induce defense programs (1). Cell-surface pattern recognition receptors (PRRs) recognize microbe- or danger-associated molecular patterns (MAMPs or DAMPs) which activate pattern-triggered immunity (PTI) (2). PTI is a low-level immune response that prevents nonadapted microbes from colonization. Host-adapted (virulent) pathogens deliver virulence factors (called effectors) which manipulate plant cells and suppress PTI, thereby facilitating infection (3). A major immune barrier to disease caused by such microbes is mediated by intracellular nucleotide-binding/leucine-rich repeat (NLR) receptors which detect particular effectors or their activities to induce effector-triggered immunity (ETI) (4). ETI is an amplified immune response involving the transcriptional boosting of PTI defenses and often localized host cell death at pathogen infection sites (1, 5).

Pathogen-detecting NLR receptors, referred to as sensor NLRs, are divided into two major functional classes defined by their N-terminal signaling domains: a coiled-coil (CC) domain in CC-NLRs (CNLs) and a Toll-Interleukin-1 Receptor (TIR) domain in TIR-NLRs (TNLs) (4). Recent studies showed that pathogen-activated sensor NLRs of both types can form signaling-active oligomeric complexes called resistosomes, which are, in principle, similar to pathogen induced NLR inflammasomes conferring innate immunity and cell death in mammals (4, 6). Certain plant CNL-type resistosomes transmit pathogen recognition autonomously to defense pathways (7, 8). Other sensor CNLs require a network of downstream oligomerizing “helper” CNLs to induce pathogen resistance and host cell death (9–13). An emerging biochemical function of sensor and helper CNL resistosomes is as Ca^2+^permeable ion channels associated with the plasma membrane (8, 13–16). Increased Ca^2+^ influx into the cytoplasm mediated by CNL- or CNL-like resistosomes is proposed to stimulate Ca^2+^ decoding systems and nuclear transcription factors which reprogram cells and tissues for defense (17, 18). However, the steps downstream of activated sensor and helper CNL resistosomes in executing immunity and cell death remain obscure.

TNL-type sensor NLRs in dicotyledenous (dicot) plants combat diverse pathogens (19). Pathogen effector-activated sensor TNL resistosomes resistance to *peronospora parasitica* (RPP1) in *Arabidopsis thaliana* (20) and recognition of XopQ1 (Roq1) in *Nicotiana benthamiana* (21), as well as pathogen-responsive TIR-only proteins (22–25), signal as NAD^+^ hydrolyzing enzymes producing cyclic and noncyclic ribosylated nucleotide products. The TNL/TIR-only generated noncyclic nucleotide signals pRib-AMP/pRib-ADP and ADPr-ATP/di-ADPR are plant immune second messengers which, respectively, activate enhanced disease susceptibility 1 (EDS1)—phytoalexin-deficient 4 (PAD4) and EDS1—senescence-associated gene 101 (SAG101) dimeric receptors (26, 27). Nucleotide-bound EDS1-PAD4 and EDS1-SAG101 dimers are recognized by a small conserved group of helper NLRs (called RNLs) with an N-terminal CC-like 4-helical bundle HET-S/LOP-B (HeLo) (CC_HeLo_ or CC_R_) signaling domain (16, 28, 29). *Arabidopsis* pRib-AMP/pRib-ADP bound EDS1-PAD4 dimers interact specifically with activated disease resistance 1 (ADR1) family RNLs, and ADPr-ATP/di-ADPR bound EDS1-SAG101 dimers with N requirement gene 1 (NRG1) family RNLs, as two distinct immune signaling modules (26, 27, 30–34). In both modules, EDS1 dimer binding results in RNL conformational activation through a similar mechanism as pathogen effector activation of sensor CNLs and TNLs, except that RNLs recognize host small molecule-modified EDS1 complexes (35–38). In vivo evidence points to ADR1- and NRG1-type RNL proteins forming CNL-like resistosomes with Ca^2+^ ion channel activity at the plasma membrane (15, 16, 39). A number of in vivo check points in helper NLR resistosome assembly have been observed as potentially important constraints on activity (13, 37–39). If CNLs and TNLs (the latter via RNLs) converge on Ca^2+^ influxes into cells, a further question remains whether and how NLR-assembled ion channels are coordinated with canonical Ca^2+^ ion channels that regulate plant immune responses and homeostasis (17, 18).

In *Arabidopsis*, signaling via the EDS1-PAD4-ADR1 or EDS1-SAG101-NRG1 module varies for different sensor TNLs, probably due to availability of their stimulating nucleotides (4). In *N. benthamiana*, sensor TNL Roq1 recognition of *Xanthomonas euvesicatoria* (*Xe*) 85-10 outer protein Q (XopQ) promotes transcriptional reprogramming, pathogen resistance, and host cell death in leaves almost exclusively via the EDS1-SAG101-NRG1 module (40–42), with the EDS1-PAD4-ADR1 module stimulating stomatal defenses (43). Here, we have exploited the *Nb*Roq1-XopQ system to explore processes in ETI execution downstream of activated EDS1-SAG101-NRG1. In a transcriptome analysis we identified a pair of glutamate receptor–like (GLR) Ca^2+^ ion channel proteins GLR2.9a and GLR2.9b which, unlike most GLRs that are mobilized in PTI, are further strongly up-regulated in the *Nb*Roq1 ETI response. By testing NRG1 structure-guided variants, we establish that an EDS1-SAG101-activated NRG1 resistosome with Ca^2+^ ion channel activity is necessary for *GLR2.9a* and *GLR2.9b* transcriptional induction in TNL-triggered ETI. We further show that both GLRs, although with different subcellular accumulation profiles, contribute to increased intracellular Ca^2+^ and to pathogen restriction and cell death in Roq1-mediated ETI. Therefore, GLR Ca^2+^ ion channels link TNL-activated EDS1-SAG101-NRG1 mediated transcription to immunity and cell death.

## Results

### EDS1-SAG101-NRG1 Control of TNL-Triggered Transcriptional Reprogramming in *N. benthamiana*.

To examine immune-related gene expression changes controlled by the EDS1-SAG101-NRG1 node in *N. benthamiana* (*Nb*), we first compared TNL (Roq1) induced cell death and disease resistance phenotypes of *Nb* wild-type (WT) and previously characterized *Nb nrg1-5* (*nrg1-5*) single (44) and *Nb eds1 pad1 sag101a sag101b* (*epss*) quadruple (30) mutants with two newly generated independent sextuple *Nb epssna* mutant lines (ln1 and ln2) (*SI Appendix*, Fig. S1*A*). Conductivity (ion leakage) assays in leaves of these lines at 24 h post infiltration (hpi) with *Pseudomonas fluorescens* 0-1 (45) (*Pf*0-1) delivering *Xe* effector XopQ (recognized by *Nb* Roq1) (46) were used to quantify host cell death. Pathogen resistance assays were performed by counting *Xe* bacteria in leaves at 6 days post infiltration (dpi). The *epss* and *epssna* mutants were equally defective in promoting Roq1-triggered cell death and pathogen resistance (*SI Appendix*, Fig. S1 *B* and *C*). These data support cooperative functions of EDS1-SAG101 dimers with NRG1 and EDS1-PAD4 dimers with ADR1 in *Nb* immunity signaling. The *Nb nrg1-5* single mutant displayed strongly reduced host cell death and full loss of bacterial resistance (*SI Appendix*, Fig. S1*B*), consistent with a major role of the EDS1-SAG101-NRG1 node and minor contribution of the EDS1-PAD4-ADR1 node in *Nb* TNL-triggered immunity in leaves (41).

**Fig. 1.**
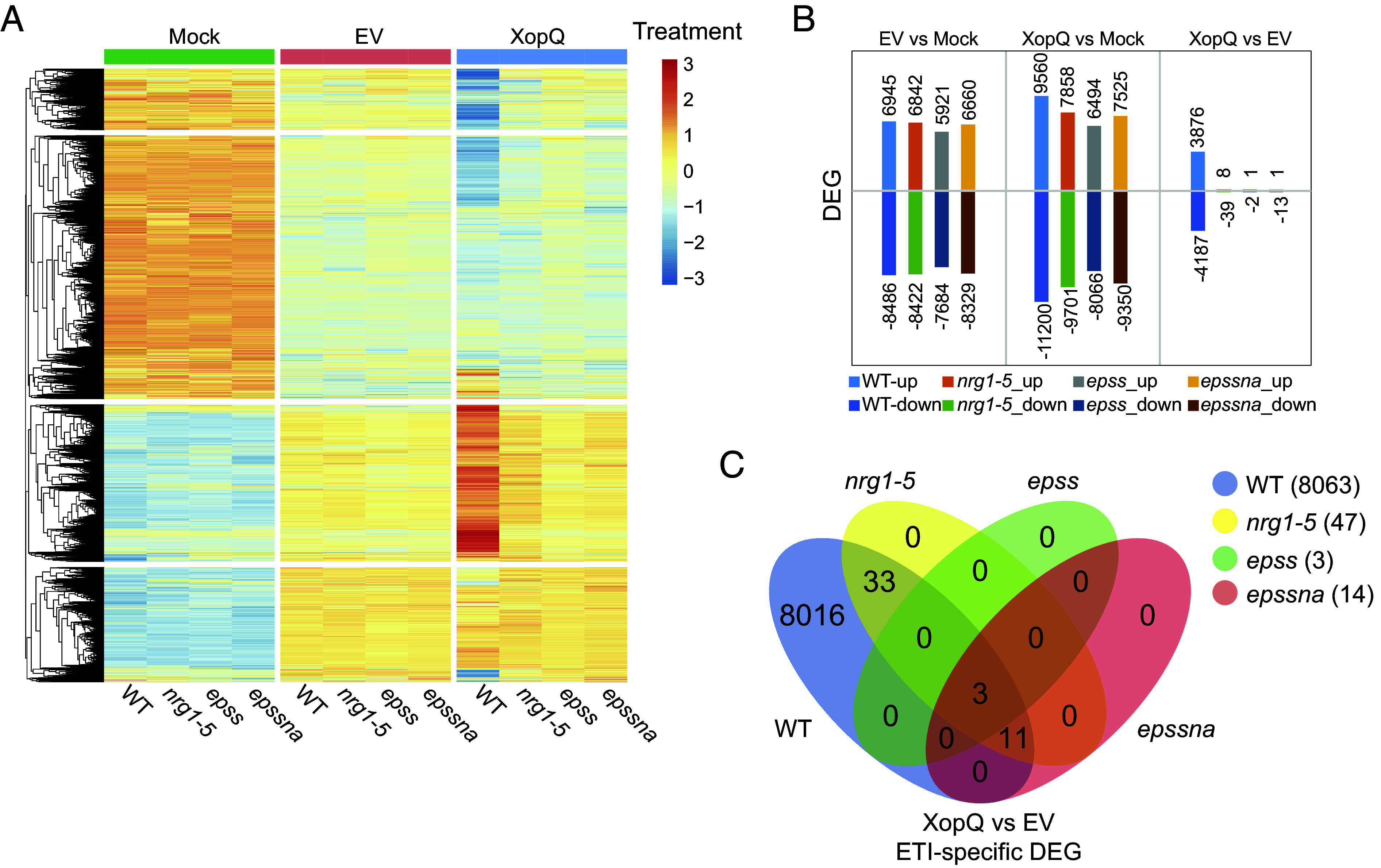
Analysis of PTI- and ETI-associated transcriptional changes and their dependence on the EDS1-SAG101-NRG1 node in *N. benthamiana*. (*A*) A heatmap representation of transcripts changes in different *Nb* genotypes, as indicated, at 6 h post MgCl_2_ (mock), *Pf*0-1 EV (EV), or *Pf*0-1 *XopQ* (XopQ) infiltration. Transcript values relative to mean normalized counts of all RNA-seq samples are shown on the scale of red (higher than the mean), yellow (close to the mean), and blue (lower than the mean). See *Materials and Methods* for details. (*B*) Total numbers of up- and down- DEG in RNA-seq samples (EV vs. mock, XopQ vs. mock, XopQ vs. EV) as indicated. DEG were selected using |log2FC| ≥ 1 and FDR < 0.05 criteria. (*C*) A Venn diagram showing overlaps between total DEG in *Nb* WT (XopQ vs. EV [8063]), *nrg1-5* (XopQ vs. EV [47]), *epss* (XopQ vs. EV [3]), and *epssna* (XopQ vs. EV [14]).

We next infiltrated leaves of *Nb* WT, *nrg1-5*, *epss,* and *epssna*_ln1 with a mock solution (10 mM MgCl_2_), *Pf*0*-*1 containing an empty vector (*Pf*0-1 EV) to elicit PTI, or *Pf*0-1 delivering XopQ to elicit TNL (Roq1) ETI, and processed samples for RNA-sequencing (RNA-seq) analysis at 6 hpi (*SI Appendix*, Fig. S1*D*). The 6 hpi time point was chosen to capture early TNL-triggered defense gene expression changes after initial detection of TNL-induced EDS1-SAG101-NRG1 association at 4 hpi with *Pf*0-1 *XopQ* (31). A principal component analysis (PCA) of three replicates for each treatment showed that mock treatments in all lines clustered together and away from the PTI and ETI treatments (*SI Appendix*, Fig. S1*E*). The *Nb* WT response to *Pf*0-1 *XopQ* (ETI), but not to *Pf*0-1 EV (PTI), separated from that of the mutants (*SI Appendix*, Fig. S1*E*), indicating that WT strongly differentiates from the *nrg1-5*, *epss,* and *epssna* mutant lines in TNL ETI but not PTI responses at 6 hpi. The RNA-seq data, represented as a heatmap of scaled gene expression values (Fig. 1*A*) or as total numbers of differentially expressed genes (DEG) per genotype (Fig. 1*B*), further showed that the EDS1-SAG101-NRG1 node promotes gene expression changes in ETI but not in PTI. Transcripts of *EDS1a*, *EDS1b*, *SAG101a,* and *NRG1* genes comprising the EDS1-SAG101-NRG1 signaling module (40), but not EDS1-PAD4-ADR1 module components *PAD4* and *ADR1*, were among strongly up-regulated genes in *Pf*0-1 *XopQ*-treated WT leaves (*SI Appendix*, Fig. S1*F*).

Further comparison of WT and mutant up- and down- regulated genes (up and down DEG) showed that *nrg1-5* retained a low number (47) of DEG compared to WT (8063) in ETI vs. PTI tissues (|log2FC| ≥1 and false discovery rate (FDR) < 0.05) which were lost in the *epss* and *epssna* mutants, as represented in a Venn diagram (Fig. 1*C*). These residual DEG in *nrg1-5* we attributed to EDS1-PAD4-ADR1 regulation of TNL ETI. Of eight ETI-specific up-DEG in *nrg1-5* (Dataset S1), WRKY-family transcription factor *NbWRKY40e* was reported to bind the *Isochorismate synthase 1* (*NbICS1*) promoter leading to increased salicylic acid biosynthesis and defense amplification impacting stomatal immunity (43, 47). Another *WRKY* gene, *NbWRKY55*, is a homolog of *AtWRKY70* which confers resistance to *Pseudomonas syringae* infection through cooperation with *AtWRKY46* and *AtWRKY53* (48, 49). These data indicate that the EDS1-SAG101-NRG1 node is chiefly responsible for TNL-triggered transcriptional defense leading to host cell death and bacterial resistance in *Nb* leaves and that the EDS1-PAD4-ADR1 node compensates only to a small degree in transcriptional defense reprogramming when the EDS1-SAG101-NRG1 node is disabled in *Nb* ETI.

### Boosted Expression of *GLR 2.9a and 2.9b* in *Nb* ETI.

Analysis of RNA-seq GO term categories enriched among DEG for tissues treated with *Pf*0-1 EV (PTI) and *Pf*0-1 *XopQ* (ETI) showed an enrichment of calcium-related processes (*SI Appendix*, Fig. S2 and Dataset S2), consistent with EDS1-SAG101 dimer assisted NRG1 assembly into a resistosome-like Ca^2+^ permeable ion channel for defense reprogramming and cell death in ETI (15, 16).

**Fig. 2.**
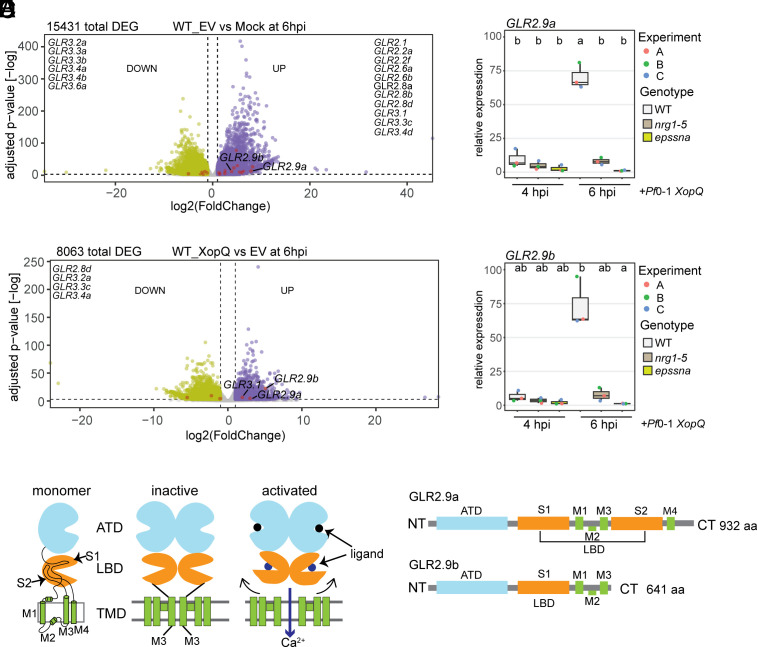
Immunity induction and functional features of *Nb*GLR2.9a and *Nb*GLR2.9b. (*A*) Volcano plot showing transcripts (DEG) responsive to *Pf*0-1 EV (PTI) in *Nb* WT (|log2FC| ≥ 1 and FDR < 0.05 for EV vs. Mock). (*B*) Volcano plot showing transcripts (DEG) responsive to *Pf*0-1 *XopQ* (ETI) in *Nb* WT (|log2FC| ≥ 1 and FDR < 0.05 for XopQ vs. EV). Purple (*Right*) and green (*Left*) dots represent up- and down-DEG respectively. Differentially expressed *GLR* transcripts are highlighted in red and listed on the plots. (*C* and *D*) qRT-PCR analysis of *GLR2.9a* (*C*) and *GLR2.9b* (*D*) transcripts in *Nb* WT, *nrg1-5,* and *epssna* at 4 h and 6 h post infiltration with *Pf*0-1 *XopQ*. Samples are normalized to *Nb* WT at 4hpi with *Pf*0-1 EV (PTI). Data are from three biological replicates. The Nemenyi test with Bonferroni correction for multiple testing was applied (α = 0.05). Different lowercase letters indicate significant differences. (*E*) Schematic diagram of *At*GLR3.4 Ca^2+^ channel gating mechanism based on protein structure analysis. Monomeric *At*GLR3.4 (*Left*). Without agonist binding GLRs adopt an inactive conformation in which the transmembrane channel (green) is closed (*Middle*). It is proposed that bound agonists (blue and black circles) to ATD (blue) and LBD (orange) induce conformational changes (black arrows) leading to ion channel opening and Ca^2+^ influx (purple arrow) (*Right*). TMD, transmembrane domain; S1 and S2 (segment 1 and segment 2); M1 to M4 (membrane-spanning domains 1 to 4), (*F*) Diagram of predicted *Nb* GLR2.9a and GLR2.9b protein domains.

We found that expression of 19*Nb GLR* genes encoding a family of canonical Ca^2+^-permeable transmembrane ion channels (50) changed in an EDS1-SAG101-NRG1 independent manner in *Pf*0-1 EV (PTI) vs. mock treatments (Fig. 2*A*). Therefore, the PTI response in *Nb* involves a general mobilization of GLR Ca^2+^ channel activities, consistent with earlier observations in *Arabidopsis* (51). The expression of *GLR* genes *NbGLR2.9a*, *NbGLR2.9b,* and *NbGLR3.1* was further significantly boosted by the EDS1-SAG101-NRG1 module in ETI, as indicated in *Pf*0-1 *XopQ* vs. *Pf*0-1 EV treatments (Fig. 2 *A* and *B* and *SI Appendix*, Fig. S3 *A* and *B*). We reasoned that this small group of *GLR*s might therefore be recruited to amplify TNL (Roq1) immunity. To test this further, we focused analysis on *NbGLR2.9a* and *NbGLR2.9b* because these genes were most prominent among the TNL (Roq1) ETI-related *GLRs* (Fig. 2*B* and *SI Appendix*, Fig. S3 *A* and *B*). Also, *NbGLR2.9a* and *NbGLR2.9b*, but not *NbGLR3.1,* were among *NRG1*-dependent induced genes in a previous study (41). qRT-PCR analysis of *Nb* WT, *nrg1-5,* and *epssna Pf*0-1 *XopQ* infiltrated leaves at 4 and 6 hpi showed that the EDS1-SAG101-NRG1 node is the major, but not exclusive, driver of induced *NbGLR2.9a* and *NbGLR2.9b* expression in TNL-triggered immunity (Fig. 2 *C* and *D*). We further noted that induced expression of *NbPR5* in ETI was *EDS1*-family but not *NRG1* dependent (*SI Appendix*, Fig. S3*C*), consistent with an EDS1-PAD4-ADR1 contribution to *Nb* defense (41).

In animals, ionotropic glutamate receptors (iGluRs) are ion channel pores activated by glutamate binding to mediate excitatory neurotransmission in the central nervous system (52). In plants, *At*GLR3.3 was shown to have a role in amino acid-induced cytosolic Ca^2+^ accumulation and is a key player in the glutamate-mediated wound response (53–55). iGluRs are composed of four subunits, each consisting of an extracellular amino-terminal domain (ATD) and ligand-binding domain (LBD), a transmembrane domain (TMD), and an intracellular carboxyl-terminal (CT) domain (52). The LBD consists of two segments, S1 and S2, which together create a clamshell-like structure around the ligand. Agonist binding to the LBDs typically drives conformational activation of iGluRs and plant GLRs (52, 56). In *Arabidopsis*, the best characterized GLRs, *At*GLR3.3, and *At*GLR3.4, exhibit permeability to Ca^2+^ upon binding of glutamate or other amino acids to the LBD (53, 54, 56) (Fig. 2*E*). The *At*GLR3.4 tetrameric structure (PDB: 7LZH) resolved by cryogenic electron microscopy (cryo-EM) revealed that its ATDs bind glutathione (GSH), which further promotes conformational changes necessary for GLR3.4 Ca^2+^ channel activity (56). The *At*GLR3.4 TMD consists of transmembrane helices M1, M3, and M4 and a reentrant intracellular loop of M2 and M3 helices forming a gate for regulated Ca^2+^ transport (Fig. 2*E*) (56). Thus, *At*GLR3.4 has structural and functional similarities to animal iGluRs (52, 56).

Based on the Sol Genomics Network Annotation file (57), the *Nb* genome encodes 36 *GLR* genes, none of which have been functionally characterized (*SI Appendix*, Fig. S4). BLASTP analysis with *Nb*GLR2.9a and *Nb*GLR2.9b in Phytozome gave *At*GLR2.9 as the closest hit. We performed a phylogenetic analysis using the full-length amino acid sequences of GLRs from *Arabidopsis* and *Nb*, and found that the closest GLRs in *Arabidopsis* are “*At*GLR2.8 and *At*GLR2.9,” based on tree-distance calculations. However, *Nb*GLR2.9a and *Nb*GLR2.9b did not map to a direct *Arabidopsis* counterpart (*SI Appendix*, Fig. S4). We therefore think it likely that *Nb*GLR2.9a/b form a group of GLRs with no clearly identifiable orthologs in *Arabidopsis*. In this study, we have kept the accepted genome annotation of *Nb*GLR2.9a and *Nb*GLR2.9b from the Sol Genomics Network to avoid confusion.

Both *Nb*GLR2.9a and *Nb*GLR2.9b possess a putative ATD, LBD, and TMD as in *At*GLR3.4. However, GLR2.9b which is shorter than GLR2.9a, lacks ATD M4 helices and the LBD S2 region that stabilizes ligand (serine, glutamate, or methionine) binding to *At*GLR3.4 (56) (Fig. 2*F*). The predicted *Nb*GLR2.9b protein sequence has 92.8% identity to *Nb*GLR2.9a (Fig. 2*F*). Modeling of *Nb*GLR2.9a and *Nb*GLR2.9b onto the active *At*GLR3.4 tetramer (56) showed that both *Nb*GLR2.9 proteins are predicted to form a similar tetrameric assembly (*SI Appendix*, Fig. S5 *A* and *B*) (56). Protein sequence alignments and structural modeling showed that, although *Nb*GLR2.9b lacks part of the LBD S2 region and TMD M4 helices, key binding residues for agonists in the LBD S1 region are shared with *Nb*GLR2.9a and *At*GLR3.4 (Fig. 2*F* and *SI Appendix*, Fig. S5*C*). Moreover, the extracellular portion of the active tetrameric *At*GLR3.4 ion conduction pore lined by M3 helices (aa 671 to 708) and gate-forming residues (56) are conserved in *Nb*GLR2.9a and *Nb*GLR2.9b (*SI Appendix*, Fig. S5*C*). These results are consistent with *Nb*GLR2.9a and *Nb*GLR2.9b both being Ca^2+^ permeable ion channels.

Collectively, the data show that *NbGLR2.9a* and *NbGLR2.9b* are transcriptionally induced by the TNL-triggered EDS1-SAG101-NRG1 module and their encoded proteins have features of agonist-gated Ca^2+^ permeable ion channels.

### An NRG1 Oligomeric Ca^2+^ Ion Channel Drives *GLR2.9a* and *GLR2.9b* Induced Expression.

**Fig. 3.**
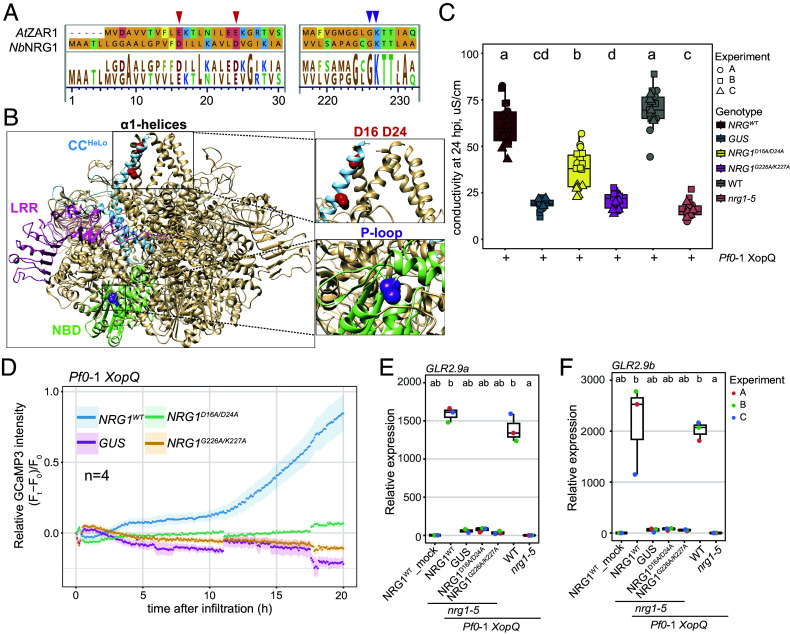
*Nb*NRG1-mediated calcium influx is necessary for XopQ-triggered host cell death and *GLR2.9a*/*GLR2.9b* up-regulation. (*A*) Alignment of *Nb*NRG1 and *At*ZAR1 at the N-terminal α1-helix and P-loop region. *Nb*NRG1 amino acids D16 and D24 aligned with residues E11 and E18 in *At*ZAR1 are indicated with red arrow heads. The conserved *Nb*NRG1 G226 K227 P-loop motif is indicated with purple arrow heads. (*B*) *Nb*NRG1 resistosome modeled on the *At*ZAR1 pentamer structure (PDB: 6J5T). *Upper Right*: Magnified α1-helical funnel with amino acids D16 and D24 in red. *Bottom Right*: Magnified P-loop region with amino acids G226 and K227 in purple. (*C*) Conductivity measurements of Roq1 triggered cell death in *Nb* WT*, nrg1-5,* and stable transformant lines of *nrg1-5*, as indicated (n = 18 from three independent experiments). Different lowercase letters indicate significant differences by one-way ANOVA followed by the post hoc test (α = 0.05). (*D*) Ribbon plots show time-course analysis of relative GCaMP3 fluorescence intensity reporting cytoplasmic Ca^2+^ levels in leaves of *nrg1-5* stable transgenic lines, as indicated, after *Pf*0-1 *XopQ* infiltration (n = 4). GCaMP3-specific fluorescent signals were recorded at 5 min intervals from 0 to 20 hpi with *Pf*0-1 *XopQ*. (F_t_−F_0_): absolute change in GCaMP3 fluorescence signal at time t relative to the baseline at t = 0. (F_t_−F_0_)/F_0_: GCaMP3 fluorescence signal change relative to the baseline. (*E* and *F*) qRT-PCR analysis of *GLR2.9a*, *GLR2.9b* in *Nb* WT, *nrg1-5* transgenic lines, as indicated, at 6 h post *Pf*0-1 *XopQ* infiltration. Samples were normalized to NRG1^WT^-myc at 6 hpi mock infiltration. Data are from three biological replicates. The Nemenyi test with Bonferroni correction for multiple testing was applied (α = 0.05). Different lowercase letters indicate significant differences.

Recent in vitro and in vivo studies showed that an *At*EDS1-*At*SAG101-*At*NRG1 stable trimeric complex forms after TNL activation (37–39). It remained unclear whether the EDS1-SAG101-NRG1 trimer has a defense function or is an inactive intermediate for assembling the signaling-active NRG1 pentamer. We therefore tested whether assembly of the *Nb*EDS1-*Nb*SAG101-activated *Nb*NRG1 resistosome with Ca^2+^ permeable ion channel activity is necessary for transcriptional up-regulation of *GLR2.9a* and *GLR2.9b* in TNL mediated ETI. By modeling *Nb*NRG1 onto the *At*ZAR1 CNL pentameric resistosome (PDB: 6J5T) (7), we identified and mutated equivalent residues (*Nb*NRG1^D16A/D24A^) to *At*ZAR1^E11A/E18A^ (Fig. 3 *A* and *B*). These amino acids are located at the inner surface of an N-terminal α1-helical funnel formed by the resistosome CC-domains and are essential for *At*ZAR-mediated Ca^2+^ influx into plant cells (14) but not *At*ZAR1 oligomerization or plasma membrane association (7, 14). Additionally, we made an *Nb*NRG1^G226A/K227A^ ADP/ATP-binding (GKT, p-loop) mutant in the *Nb*NRG1 NB-domain to disable oligomerization (Fig. 3 *A* and *B*) (16, 31).

We tested the functionality of native promoter-driven C-terminally 4x myc-tagged NRG1^WT^, NRG1^D16A/D24A^, and NRG1^G226A/K227A^ in an *Nb epssna* transient reconstitution assay for TNL (Roq1) resistance to *Xe* bacteria. The NRG1 proteins were each coexpressed with *Nb*EDS1a-FLAG and *Nb*SAG101b-GFP (or GFP as control) by agroinfiltration. *Xe* was coinfiltrated into *Nb* leaf sectors and *Xe* bacterial growth measured at 6 dpi. Both NRG1^D16A/D24A^ and NRG1^G226A/K227A^ failed to limit *Xe* whereas NRG1^WT^ restricted *Xe* infection, although not as strongly as WT plants (*SI Appendix*, Fig. S6*A*). This is consistent with a previous finding that transient expression of the *At* EDS1-SAG101-NRG1 module does not fully complement resistance (30). The NRG1^D16A/D24A^ and NRG1^G226A/K227A^ variants transiently expressed in *nrg1-5* leaves also failed to promote XopQ-triggered cell death compared to NRG1^WT^ (*SI Appendix*, Fig. S6*B*). These experiments demonstrated that an NRG1 C-terminal 4× myc-tag does not impair its TNL-triggered immunity function, whereas disabling EDS1-SAG101-dependent NRG1 oligomerization and ion channel activity does disrupt its function.

We next made stable transgenic lines expressing the *pNRG1: NRG1-myc*, *pNRG1:NRG1^D16A/D24A^-myc*, *pNRG1:NRG1^G226A/K227A^-myc* transgenes or *pNRG1:GUS-myc* (as negative control) in *nrg1-5* and selected individual lines with similar NRG1-myc protein accumulation for analysis (*SI Appendix*, Fig. S6*C*). While NRG1^WT^ rescued *Pf0*-1 *XopQ* triggered cell death in *nrg1-5*, NRG1^D16A/D24A^ showed a partial and NRG1^G226A/ K227A^ a complete loss of rescue (Fig. 3*C*).

To compare NRG1^WT^ and the two NRG1 mutant variants in TNL (Roq1) ETI-stimulated calcium influx into cells, we transiently expressed a fluorescent Ca^2+^ sensor GCaMP3 (13, 58) in the *nrg1-5* complementation lines and infiltrated *Pf*0-1 *XopQ* into the same leaf sectors after 2 d to elicit ETI. NRG1^WT^ produced a steady increase in [Ca^2+^]_cyt_ from 4 to 5 hpi corresponding to the timepoint when EDS1-SAG101-NRG1 association is detected in IP experiments (31, 39, 59). This was followed by a steeper rise in Ca^2+^from ~10 to 20 hpi which we attributed to progression of cell death (31). By contrast, the NRG1^G226A/K227A^ p-loop mutant and the GUS negative control exhibited no early (3 to 6 h) or late (from 10 h) Ca*^2+^* increase. The NRG1^D16A/D24A^ mutant exhibited strongly reduced Ca*^2+^* influx from 4 hpi (Fig. 3*D*). *GLR2.9a* and *GLR2.9b* mRNA levels measured by qRT-PCR at 6 hpi with *Pf*0-1 *XopQ* showed that NRG1^WT^ induced *GLR2.9a* and *GLR2.9b* up-regulation, whereas *Nb*NRG1^D16A/D24A^ and *Nb*NRG1^G226A/K227A^ were defective in promoting expression of these genes (Fig. 3 *E* and *F*). Put together, these results suggest that an EDS1-SAG101-activated NRG1 resistosome with ion channel activity, and not the EDS1-SAG101-NRG1 trimer, is responsible for transcriptional induction of *GLR2.9a* and *GLR2.9b* in *Nb* TNL triggered immunity.

### GLR2.9a and GLR2.9b Contribute to TNL-Triggered Cell Death and Pathogen Resistance.

**Fig. 4.**
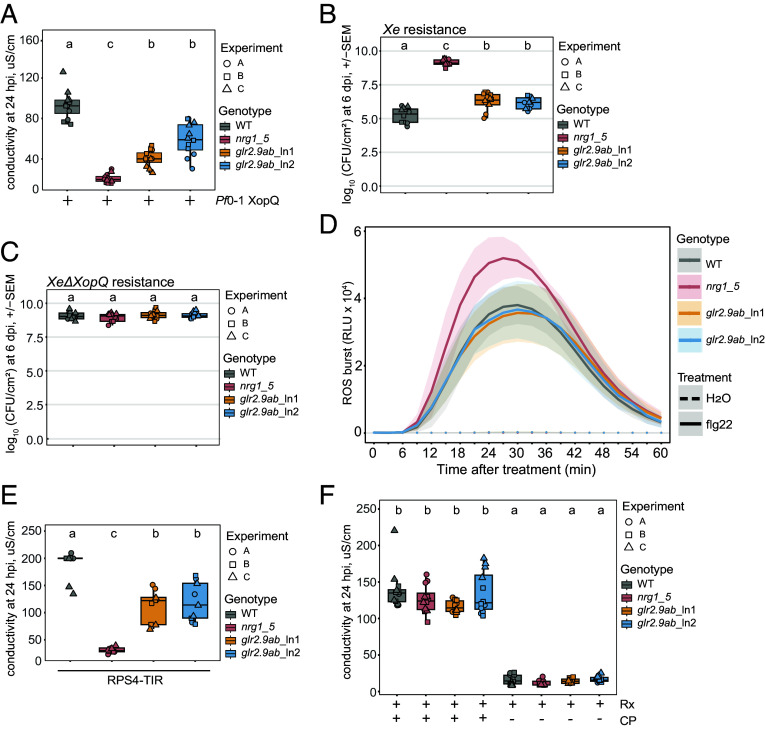
*Nb*GLR2.9a and *Nb*GLR2.9b promote TNL Roq1 triggered host cell death and pathogen resistance. (*A*) Conductivity measurements of Roq1-mediated cell death in *Nb* WT*, nrg1-5*, *glr2.9ab*_ln1, and *glr2.9ab*_ln2 at 1 d with *Pf*0-1 *XopQ* (n = 12 from three independent experiments). (*B*) *Xe* growth assay in leaves of the same lines as (*A*) at 6 dpi with *Xe* (n = 12 from three independent experiments). (*C*) *Xe*Δ*XopQ* growth assays in leaves of the same lines as (*A*) at 6 dpi (n = 12 from three independent experiments). (*D*) *Nb* WT*, nrg1-5*, *glr2.9ab*_ln1, and *glr2.9ab*_ln2 show comparable ROS production in response to 1 μm flg22 treatment. ROS production was monitored using a chemiluminescence assay. Data are presented as average signals from three independent experiments. Ribbon plots represent mean value ± SE (n = 8 biologically independent discs) with all individual data points. (*E*) Conductivity measurements of RPS4 TIR triggered cell death in leaves of the same lines as (*A*) at 24 h post agroinfiltration (n = 9 from three independent experiments). (*F*) Conductivity measurements of CNL Rx triggered cell death in leaves of the same lines as (*A*) at 24 h post agroinfiltration of Rx with or without PVX coat protein effector (CP) (n = 12 from three independent experiments). (*A–F*) Genotypes with different letter codes are significantly different. The one-way ANOVA followed by the post hoc test was applied (α = 0.05).

We used CRISPR-Cas9 mutagenesis to assess whether *GLR2.9a* and *GLR2.9b* are required for TNL-triggered immunity in *Nb*. Two small guide (sg)RNAs were designed to target each of the *GLR2.9a* and *GLR2.9b* genes simultaneously in *Nb* WT and two independent Cas9-free *glr2.9ab* homozygous double mutant lines (ln1 and ln2) were selected (*SI Appendix*, Fig. S7*A*). We compared TNL (Roq1) ETI responses of *glr2.9ab* plants to those of *Nb* WT and *nrg1-5*. Host cell death elicited in leaves after infiltration of *Pf*0-1 *XopQ* was again quantified at 1 d by conductivity assays. The *glr2.9ab* mutants showed a partial loss of cell death, lying between WT and *nrg1-5* plants (Fig. 4*A*). In TNL Roq1 resistance assays to *Xe* delivering XopQ, restriction of bacterial growth at 6 dpi in leaves of *glr2.9ab* was also intermediate between *Nb* WT and *nrg1-5* (Fig. 4*B*). By contrast, growth of a nonrecognized *XopQ* deletant strain of *Xe* (*Xe*Δ*XopQ*) was unaffected in *glr2.9ab* and *nrg1-5* mutants (Fig. 4*C*). We measured responses to the bacterial PAMP flg22 in *Nb* WT, *glr2.9ab,* and *nrg1-5* plants and found that a flg22-induced ROS burst and expression of *CYP71D20* (a PTI marker gene in *Nb*) (60) were similar between WT and mutant plants (Fig. 4*D* and *SI Appendix*, Fig. S7 *B* and *C*). Hence, *GLR2.9a* and *GLR2.9b* are required for full TNL-triggered, NRG1-driven ETI but not for basal immunity or flg22-triggered immune responses in *Nb*.

Additionally, transient overexpression of the TIR domain from *Arabidopsis* TNL receptor Resistance to *Pseudomonas syringae* 4 (RPS4), which has NADase- and EDS1-SAG101-NRG1-dependent cell death activity in *Nb* (26, 61), produced lower cell death in the *glr2.9ab* mutants compared to WT *Nb* (Fig. 4*E*). Therefore, *GLR2.9a* and *GLR2.9b* can stimulate *Nb* defenses downstream of TNL and TIR NADases. The *glr2.9ab* mutants were not compromised in cell death elicited by a CNL-class sensor NLR potato Rx activated by its coexpression with Rx-recognized potato virus X coat protein (CP) (Fig. 4*F*) (62) or by the barley CNL receptor MLA13 activated by effector AVR_A13_-1 (63) in *Nb* leaves (*SI Appendix*, Fig. S7*D*). These data suggest that *GLR2.9a* and *GLR2.9b* are downstream components specifically of the TNL- or TIR-triggered EDS1-SAG101-NRG1 node to promote host cell death and pathogen restriction in *Nb*.

### GLR2.9a and GLR2.9b Display Different Subcellular Locations.

**Fig. 5.**
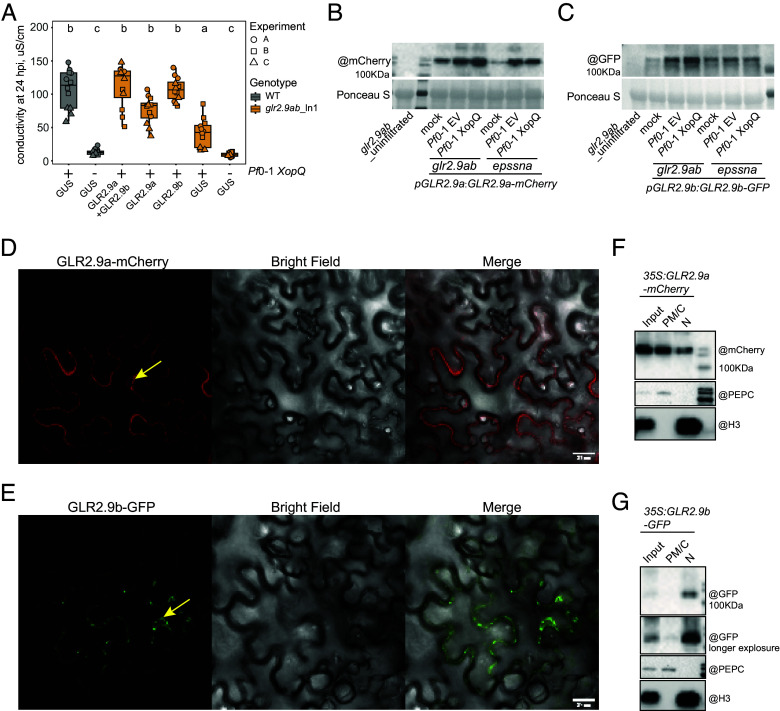
GLR2.9a and GLR2.9b proteins display different subcellular distributions. (*A*) Transient expression of GLR2.9a-mCherry together with GLR2.9b-GFP and GLR2.9a-mCherry or GLR2.9b-GFP alone, but not GUS-GFP, can rescue XopQ triggered cell death in *glr2.9ab*_ln1. At 2 d post agroinfiltration of the constructs, *Pf0*-1 *XopQ* was infiltrated into the same leaf sectors and samples were collected at 6 hpi for conductivity measurements. *Nb* WT infiltrated with or without *Pf*0-1 *XopQ* serves as positive and negative control (n = 12 from three independent experiments). Lowercase letters indicate significant differences measured by one-way ANOVA followed by the post hoc test (α = 0.05). (*B* and *C*) Western blot analysis of GLR2.9a-mCherry (*B*) and GLR2.9b-GFP (*C*) protein accumulation in *Nb glr2.9ab_*ln1 or *Nb epssna* leaves at 6 h post treatment with MgCl_2_ (mock), *Pf0*-1 EV, or *Pf*0-1 *XopQ*. GLR2.9a-mCherry and GLR2.9b-GFP proteins were detected using α-mCherry and α-GFP antibodies, respectively. Three independent experiments showed the same trends. (*D* and *E*) Subcellular localizations of constitutively expressed GLR2.9a-mCherry (*D*) and GLR2.9b-GFP (*E*) at 2 d post agroinfiltration into *Nb glr2.9ab* leaves, imaged by confocal microscopy and analyzed in three independent experiments (five cells imaged/experiment). Representative images of GFP (green) and mCherry (red) signals are shown. Yellow arrows point to the nucleus. (Scale bar, 25 μm.) (*F* and *G*) Isolation of total (Input), plasma membrane/cytoplasm (PM/C), and nuclear (N) enriched fractions at 2 d post infiltration of *Nb glr2.9ab* leaves with 35S:GLR2.9a-mCherry (*F*) and 35S:GLR2.9b-GFP (*G*). α-PEPC and α-Histone H3 were used as cytosolic and nuclear markers, respectively. Three independent experiments showed similar results.

In transient expression assays we tested whether fluorescent protein-tagged *Nb*GLR2.9a and *Nb*GLR2.9b expressed under control of their respective native promoter (*pGLR2.9a:gGLR2.9a-mCherry* and *pGLR2.9b:gGLR2.9b-GFP*) could rescue *Pf*0-1 *XopQ*-triggered cell death in *glr2.9ab* ln1 (Fig. 5*A*). In these assays expression of GLR2.9a-mCherry and GLR2.9b-GFP alone or together by agroinfiltration did not induce cell death at 2 dpi (*SI Appendix*, Fig. S7*E*), after which *Pf*0*-1 XopQ* was infiltrated into leaf sectors to activate TNL Roq1. GLR2.9a-mCherry and GLR2.9b-GFP individually or coexpressed, but not GUS-GFP (*p35S:GUS-GFP*), complemented the *Nb glr2.9ab* ln1 mutant in TNL (Roq1) cell death, although GLR2.9b-GFP alone routinely produced a stronger TNL-triggered cell death response than GLR2.9a-mCherry (Fig. 5*A*). The GLR2.9a and GLR2.9b proteins were detected on a Western blot probed, respectively, with α-mCherry and α-GFP antibodies (*SI Appendix*, Fig. S7*F*). We concluded that *Nb*GLR2.9a-mCherry and *Nb*GLR2.9b-GFP are each able to rescue *Nb glr2.9ab* ln1 defects in TNL-triggered cell death to *Pf*0-1 *XopQ* and that *NbGLR2.9b* alone can fully compensate for combined loss of *GLR2.9a* and *GLR2.9b*.

We monitored GLR2.9a-mCherry or GLR2.9b-GFP protein accumulation at 2 d after agroinfiltration in *Nb glr2.9ab* (ln1) or *epssna* leaves followed by 6 h mock, *Pf*0-1 EV, or *Pf*0-1 *XopQ* treatment. In the *glr2.9ab* background, levels of both GLR2.9 proteins increased in response to *Pf*0-1 EV vs. mock, and further in response to *Pf*0-1 *XopQ* vs. *Pf*0-1 EV (Fig. 5 *B* and *C*). The *Pf*0-1 *XopQ* (ETI) vs. *Pf*0-1 EV (PTI) increment in GLR2.9a and GLR2.9b protein accumulation was lost in *epssna* leaves (Fig. 5 *B* and *C*). These data suggest that GLR2.9a and GLR2.9b protein accumulation after TNL triggering is strongly EDS1-SAG101-NRG1-dependent, consistent with their respective mRNAs (Fig. 2 *C* and *D*). Increased GLR2.9a accumulation in *Pf*0-1 EV vs. mock treatments did not require EDS1-RNL signaling (Fig. 5*B*), fitting with *GLR2.9a* gene expression data (*SI Appendix*, Fig. S3*B*) and suggesting that a different mechanism underlies GLR2.9a and GLR2.9b accumulation in *Nb* PTI.

We tested the subcellular localizations of native promoter-controlled GLR2.9a-mCherry or GLR2.9b-GFP transiently expressed in *glr2.9ab* ln1 using confocal microscopy at 6 hpi mock, *Pf*0-1 EV, or *Pf*0-1 *XopQ* treatment. For GLR2.9a-mCherry, a weak signal consistent with GLR2.9a protein accumulation at the plasma membrane/cytoplasm (PM/C) compartment was detected after *Pf*0-1 EV, and a stronger signal after *Pf*0-1 *XopQ* treatment (*SI Appendix*, Fig. S8*A*). We did not detect *Nb*GLR2.9b-GFP in these assays even with a *Pf*0-1 *XopQ* stimulus (*SI Appendix*, Fig. S8*B*). We therefore used biochemical fractionation assays followed by Western blotting to monitor subcellular accumulation of the same native promoter-driven GLR2.9a-mCherry and GLR2.9b-GFP constructs in *glr2.9ab* leaves at 6 h post mock, *Pf*0-1 EV, or *Pf*0-1 *XopQ* infiltration. In *Pf*0-1 EV and *Pf*0-1 *XopQ* elicited tissues, most GLR2.9a-mCherry accumulated in the PM/C fraction with a minor nuclear-enriched pool also detected (*SI Appendix*, Fig. S8*C*). By contrast, a *Nb*GLR2.9b-GFP signal was detected predominantly in the nuclear-enriched fraction and was strongest after *Pf*0-1 *XopQ* treatment (*SI Appendix*, Fig. S8*D*). These data suggest that GLR2.9a and GLR2.9b proteins have different subcellular preferences in TNL ETI-elicited *Nb* leaf cells.

Because we could not detect transiently expressed native promoter-driven GLR2.9b-GFP in *glr2.9ab* ln1 leaves using confocal microscopy imaging (*SI Appendix*, Fig. S8*B*), we monitored fluorescence signals from GLR2.9a-mCherry or GLR2.9b-GFP transiently expressed under control of a CaMV-35S constitutive promoter in *glr2.9ab* ln1 leaves without an immune trigger. We found that GLR2.9a-mCherry localized mainly to the PM/C compartment but also to the nuclear envelope (NE), with part of NE signal also possibly coming from the endoplasmic reticulum proximal to the nucleus (Fig. 5*D*). GLR2.9b-GFP signals were detected predominantly at the NE and weakly at the PM/C, and appeared to form puncta in both compartments (Fig. 5*E*). We did not observe cell collapse or death at 2 dpi when images were taken (*SI Appendix*, Fig. S7*E*), suggesting that GLR enhanced expression alone is insufficient to elicit cell death. Overexpression of GLR2.9a and/or GLR2.9b did not rescue the loss of cell death in *nrg1-5* after Roq1 activation or in response to RPS4-TIR (*SI Appendix*, Fig. S9 *A* and *B*). Also, flg22-induced cell death in *nrg1-5* was not enhanced by GLR2.9a and/or GLR2.9b overexpression (*SI Appendix*, Fig. S9*C*). These data support the notion that up-regulated GLR2.9a and GLR2.9b do not signal without NRG1-induced defense reprogramming. Biochemical fractionation of the constitutively expressed GLR2.9a-mCherry and GLR2.9b-GFP proteins in *glr2.9ab* supported different PM/C vs. NE distributions (Fig. 5 *F* and *G*), as observed for the native promoter-driven GLR2.9a-mCherry and GLR2.9b-GFP proteins in *Pf*0-1 *XopQ*-triggered tissues (*SI Appendix*, Fig. S8 *C* and *D*). Collectively, the imaging and biochemical fractionation data suggest that while GLR2.9a and GLR2.9b are both strongly induced in TNL ETI responding tissues, they have different subcellular preferences, with GLR2.9a accumulating mainly at the PM/C and GLR2.9b at the NE.

### GLR2.9a and GLR2.9b Promote Ca^2+^ Influx into Cells in TNL-Triggered ETI.

**Fig. 6.**
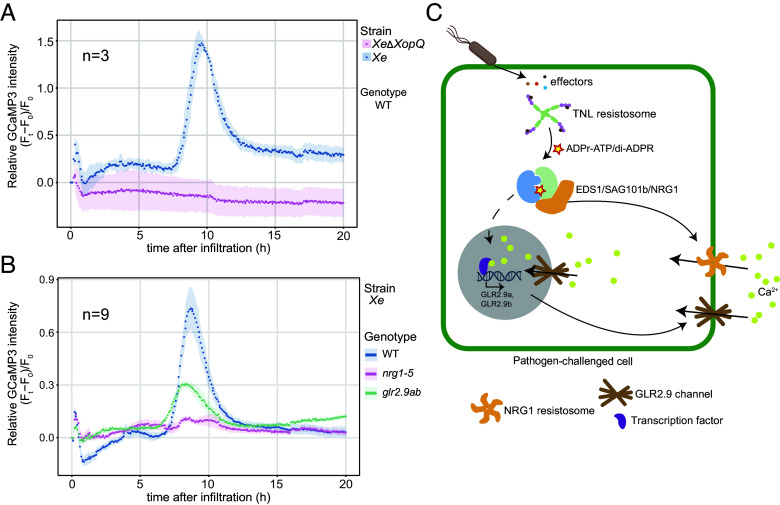
*Nb*NRG1, *Nb*GLR2.9a, and *Nb*GLR2.9b promote Ca^2+^ influx in TNL triggered ETI. (*A*) Ribbon plots show time-course analysis of relative GCaMP3 fluorescence intensity reporting cytoplasmic Ca^2+^ levels in leaves of *Nb* WT expressing GCaMP3, after *Xe*Δ*XopQ* or *Xe* infiltration (n = 3). GCaMP3-specific fluorescent signals were recorded at 5 min intervals from 0 to 20 hpi. (*B*) Ribbon plots show time-course analysis of relative GCaMP3 fluorescence intensity reporting cytoplasmic Ca^2+^ levels in leaves of *Nb* WT, *glr2.9ab,* and *nrg1-5* expressing GCaMP3 upon infiltration with *Xe*. GCaMP3-specific fluorescent signals were recorded at 5 min intervals from 0 to 20 hpi. (*C*) A working model of signaling events in *Nb* TNL triggered cell death and pathogen immunity. *Xe* delivers effector XopQ into the host cell, which is recognized by TNL Roq1. The XopQ-activated Roq1 TNL resistosome is an NADase enzyme producing ADPr-ATP and di-ADPR nucleotide small molecules which specifically bind to and promote activation of an EDS1-SAG101-NRG1 complex. The resulting NRG1 pentameric resistosome forms a Ca^2+^ permeable ion channel at the plasma membrane which promotes Ca^2+^ influx into the cell, leading to transcription factor mediated defense reprogramming in the nucleus. *GLR* genes *GLR2.9a* and *GLR2.9b* are up-regulated downstream of NRG1 resistosome ion channel activity and, through their further promotion of Ca^2+^ influx into cells, contribute to TNL effector-triggered immunity. GLR2.9a and GLR2.9b proteins accumulate at the PM and NE to confer host cell death and pathogen resistance.

To assess the contributions of GLR2.9a and GLR2.9b to NRG1-dependent calcium influx into cells in TNL triggered ETI, we crossed a previously characterized stable transgenic *Nb* WT line expressing GCaMP3 (*pCaMV35S:GCaMP3*) (58) with *nrg1-5* and *glr2.9ab* ln1. Homozygous lines were selected and used to quantify changes in cytoplasmic Ca^2+^ levels ([Ca^2+^]_cyt_). Leaves of GCaMP3 sensor-expressing *Nb* WT plants were infiltrated with Roq1-recognized *Xe* or nonrecognized virulent *Xe*Δ*XopQ* bacteria and [Ca^2+^]_cyt_ dynamics were measured over 20 h as changes in GCaMP3 fluorescence over time. These assays showed a TNL ETI-specific cytoplasmic Ca^2+^ increase between 7 and 12 hpi (Fig. 6*A*). We excluded possible interference from autofluorescence due to host cell death because *Xe* induced macroscopic cell death in these assays was first observed between 1 and 2 dpi (*SI Appendix*, Fig. S10). We next compared *Nb* WT with *nrg1-5* and *glr2.9ab* leaves after *Xe* infiltration. In contrast to the prominent Ca^2+^ peak between 7 and 12 hpi in *Xe*-elicited WT, the *glr2.9ab* mutant had reduced cytoplasmic Ca^2+^ accumulation and *nrg1-5* displayed no Ca^2+^ peak (Fig. 6*B*). We concluded that part of the EDS1-SAG101-NRG1-driven TNL ETI response in *Nb* is mediated by up-regulated GLR2.9a and GLR2.9b Ca^2+^ channel activities contributing to host cell death and pathogen resistance.

## Discussion

To investigate processes operating downstream of the EDS1-SAG101-NRG1 node in TNL (Roq1) mediated ETI, we focused analysis on *GLR2.9a* and *GLR2.9b* Ca^2+^ channel genes and their proteins. These were among numerous PTI induced (EDS1-SAG101-NRG1 independent) *GLRs* in *Nb*, indicative of a general mobilization of GLR channel activities in PTI (51, 54). *GLR2.9a* and *GLR2.9b* were exceptional in being further boosted in expression through the EDS1-SAG101-NRG1 node in TNL ETI (Fig. 2 *A* and *B*). Reduced TNL-triggered *NRG1*-dependent Ca^2+^ influxes into cells recorded by a GCaMP3 fluorescence reporter (Fig. 6*B*), as well as compromised *Xe* resistance and host cell death in *Nbglr2.9ab* mutant lines (Fig. 4 *A*, *B*, and *D*), show that the transcriptionally induced GLR2.9a and GLR2.9b Ca^2+^ ion channels contribute to ETI, as depicted in Fig. 6*C*. Therefore, EDS1-SAG101-NRG1 mobilized canonical GLR2.9a and GLR2.9b Ca^2+^ ion channel activities are an important *Nb* TNL immunity output.

We used *Nb* combinatorial mutants to assess relative contributions of the EDS1-PAD4-ADR1 and EDS1-SAG101-NRG1 modules to PTI (*Pf*0-1 *EV*) and TNL ETI (*Pf*0-1 *XopQ*) transcriptomes (Fig. 1 *A* and *B*). As previously reported (41), there was an almost exclusive requirement for the EDS1-SAG101-NRG1 node in TNL-triggered gene expression changes in *Nb* leaves, with the EDS1-PAD4-ADR1 node playing a very minor role (Fig. 1 *A* and *B*). It emerged recently that the *Nb* EDS1-PAD4-ADR1 node is important for transcriptional reprogramming in stomatal immunity (43). In our analysis, loss of both EDS1-SAG101-NRG1 and EDS1-PAD4-ADR1 signaling branches (in *Nb epss* and *Nb epssna* mutants) only mildly impaired PTI-related gene expression in response to *Pf*0-1 EV bacteria vs. a mock treatment (Fig. 1*B*). This result tallies with an earlier report that EDS1 dimers and their respective RNLs are not required for PTI signaling from tested PRRs in *Nb* leaves (42). Therefore, transcriptional mobilization of defenses including multiple *GLR* genes in PTI (Fig. 1 *A* and *B*) appears to rely on different, presumably TNL/TIR-independent, mechanisms. The observed dispensability of *GLR2.9a* and *GLR2.9b* for limiting growth of virulent *Xe*Δ*XopQ* bacteria (Fig. 4*C*) or for PAMP flg22 induced ROS burst and *CYP71D20* defense gene expression in leaves (Fig. 4*D* and *SI Appendix*, Fig. S7 *B* and *C*) might be due to redundancy with other induced *GLR*s (Fig. 2*A*) and probably other classes of Ca^2+^ ion channel, such as CNGCs which are involved in PTI and ETI regulation (17, 18).

The biochemical mechanism of NRG1 conformational activation by association with ADPr-ATP/di-ADPR bound EDS1-SAG101 was resolved in two recent structural studies of the *Arabidopsis* EDS1-SAG101-NRG1 protein complex (37, 38). These analyses identified interfaces formed between TNL-triggered EDS1-SAG101 C-terminal domains and the extreme C-terminal portion of NRG1 to promote NRG1 activation and host cell death. Assembly of an NRG1 stable pentamer required for the N-terminal α1-helix Ca^2+^ ion channel activity (15, 16) was not achieved in insect cell expression assays (37, 38). Instead, a stable EDS1-SAG101-NRG1 trimeric complex formed, which was also prominent in *Arabidopsis* tissues engineered to elicit ETI without PTI (39). Notably, accumulation of oligomeric *At*NRG1 putative resistosomes at the plasma membrane required additional PTI signals with ETI (39). Also, pathogen-induced *Arabidopsis* and *Nb* EDS1-SAG101-NRG1 protein pools were detected in nuclei (39, 42). Put together, these characteristics raised the possibility of an alternative EDS1-SAG101-NRG1 trimer function in transcriptional defense. To critically test this we examined pathogen immunity, cell death, and Ca^2+^ influx phenotypes of *Nb*NRG1 mutant variants disabled in EDS1-SAG101-mediated oligomerization (p-loop G226A/K227A) or N-terminal CC_R_ α1-helix ion channel function (D14A/D16A) (Fig. 3 *C* and *D* and *SI Appendix*, Fig. S6 *A* and *B*) (16, 31). Our data show that an *Nb*NRG1 resistosome with Ca^2+^ ion channel activity is the chief driver of *GLR2.9a* and *GLR2.9b* up-regulation (Fig. 3 *E* and *F*) and of TNL ETI resistance and host cell death (Fig. 3*C* and *SI Appendix*, Fig. S6 *A* and *B*). Whether pathogen-activated NRG1 resistosomes signal exclusively at the plasma membrane (15, 16) or more broadly at endomembrane compartments and/or the NE requires further analysis. The CC_R_ domain is present in several key membrane-associating immune regulators with different architectures to NLRs (29, 64). *Arabidopsis* CC_R_ containing mixed-lineage kinase domain-like (MLKL 1) protein contributes to TNL-activated *EDS1*-dependent immune potentiation in leaves and, as part of this process, forms puncta at the plasma membrane (59, 65). The MLKL N-terminal CC_R_ domains within an activated tetramer mediated sustained Ca^2+^ influx into cells (59), thus adding to the network of canonical and noncanonical ion channels orchestrating plant immune responses (17, 34).

Emerging evidence suggests that plant-specific factors or processes provide check points in the assembly of signaling-active helper NLR resistosomes (13, 37–39), probably to prevent undesirable spread of defenses and cell death. A similar principle might apply to the GLR2.9a and GLR2.9b ion channels because their transient overexpression in *Nb* leaves without an immune trigger did not elicit cell death (*SI Appendix*, Fig. S7*E*). Also, overexpressed GLR2.9a and/or GLR2.9b failed to alleviate the loss of TNL (Roq1) or TIR (RPS4)-triggered cell death in *nrg1-5* (*SI Appendix*, Fig. S9 *A* and *B*). We conclude that other signals induced by the EDS1-SAG101-activated NRG1 resistosome converge on up-regulated GLR2.9a and GLR2.9b proteins to stimulate their Ca^2+^ ion channel activities. Binding of reduced glutathione (GSH) within the ATD of *At*GLR3.4 contributed to Ca^2+^ ion channel gating (56). Also, exogenously supplied GSH promoted Ca^2+^cytosolic influx and defense gene expression in *Arabidopsis* (54). Besides GSH, glutamate and methionine were reported to bind to the LBDs of *At*GLR3.4 and *At*GLR3.3 and stimulate Ca^2+^ ion transport (56, 66). GSH provision slowed pathogen growth while glutamate enhanced the wound response to insect herbivores through an *At*GLR3.3-dependent pathway (54, 67). Although *Nb*GLR2.9b protein lacks an LBD S2 portion which in *At*GLR3.4 contains three residues (F624, E668, and Y671) for agonist binding and two residues (Q620, D621) to stabilize the ligand (56), conserved binding (D570, T572, R577) and stabilizing residues (R501, N549, Y552) present in LBD S1 might be sufficient to activate GLR2.9b (*SI Appendix*, Fig. S5*C*). Moreover, while the M4 membrane spanning segment present in structurally resolved *At*GLR3.4 and the modeled *Nb*GLR2.9a is missing in *Nb*GLR2.9b (Fig. 2*F* and *SI Appendix*, Fig. S5 *A*–*C*) (56), GLR2.9b retains key GLR ion channel functional motifs (Fig. 2*F* and *SI Appendix*, Fig. S5 *B* and *C*). Also, GLR2.9b complemented TNL-triggered cell death when transiently expressed in *Nbglr2.9ab_*ln1 (Fig. 5*A*), suggesting that this shorter GLR2.9 isoform is a functional Ca^2+^ ion channel. Further investigation is required to determine how GLR2.9a and GLR2.9b ion channel activities are regulated, beyond their transcriptional induction by EDS1-SAG101-activated NRG1 in TNL mediated ETI.

Data showing where plant GLRs accumulate in cells are sparse, although a plasma membrane localization was proposed for *Arabidopsis* GLR3.4 where it can be activated by amino acids to transport Ca^2+^ into the cytoplasm (68). Our confocal microscopy imaging and subcellular fractionation assays suggest that *Nb*GLR2.9a-mCherry and *Nb*GLR2.9b-GFP accumulate at the PM/C and NE to different ratios when transiently expressed in *Nbglr2.9ab* leaves (Fig. 5 *D*–*G* and *SI Appendix*, Fig. S8). Especially striking was a prominent *Nb*GLR2.9b-GFP pool enriched with nuclei after TNL triggering of leaves (*SI Appendix*, Fig. S8*D*), whereas *Nb*GLR2.9a-mCherry accumulated preferentially at the PM/C (*SI Appendix*, Fig. S8*C*). Since overexpressed *Nb*GLR2.9a-mCherry and *Nb*GLR2.9b-GFP without an immune trigger (Fig. 5 *F* and *G*) displayed similar subcellular profiles as their native-promoter controlled counterparts in TNL immune-triggered cells (*SI Appendix*, Fig. S8 *C* and *D*), we speculate that GLR2.9a and GLR2.9b isoforms are preferentially targeted and/or stabilized, respectively, at the PM/C and NE. Puncta detected for *Nb*GLR2.9b-GFP in both compartments (Fig. 5*E*) resemble those formed at the plasma membrane by an autoactive *At*NRG1.1 D485V variant and effector-activated *At*NRG1.1 WT in plant cells (15, 16). A loss-of-oligomerization mutant *At*NRG1.1 L134E failed to produce puncta compared to *At*NRG1.1 WT and abolished TNL-triggered cell death (15). Hence, puncta might reflect biologically active RNL and GLR protein foci. Our data provide a snapshot of GLR2.9a and GLR2.9b subcellular accumulation. It remains to be tested—especially for native promoter regulated GLR2.9b-GFP which we failed to detect by fluorescence imaging even under TNL ETI conditions (*SI Appendix*, Fig. S8*B*)—whether dynamic GLR2.9 protein relocalization to particular sites occurs after their TNL-triggered upregulation. In *Medicago truncatula*, three CNGC15 ion channel members were found to localize to the NE of root cells in order to modulate nuclear Ca^2+^ release which was necessary for host accommodation of nutrient-provisioning endosymbionts (69). In the *Nb* leaf response to pathogens it is possible that GLR2.9a activity at the PM/C and GLR2.9b at the NE help to optimize cytoplasmic and nuclear Ca^2+^-dependent decoding for rapid defense.

## Materials and Methods

### Plant Accession Numbers.

*NbGLR2.9a* (Niben101Scf01212g02011); *NbGLR2.9b* (Niben101Scf08670g00020); *NbNRG1* (Niben101Scf02118g00018); *NbADR1* (Niben101Scf02422g02015).

### Plant Materials and Growth Conditions.

The *epss* and *nrg1-5* mutants are published (30, 44). *epssna In1 and ln2* and *glr2.9ab In1 and ln2* mutants were generated in this study. Guide RNA sequences were designed to target *NbGLR2.9a*, *NbGLR2.9b*, *NbNRG1,* and *NbADR1*, and the corresponding oligonucleotides were annealed and ligated into a pDONR-based entry plasmid containing the *Cas9* gene and a U6-26 promoter for guide RNA expression. An LR reaction (Life Technologies) was used to move the guide and Cas9 cassette into a Gateway compatible version of the pCambia2300 vector (70). Primers used for CRISPR Cas9 mutagenesis constructs are listed in *SI Appendix*, Table S1. Simultaneous CRISPR-Cas9 knockout mutations of *NbNRG1* and *NbADR1* were selected in the *epss* background; mutations of *NbGLR2.9a* and *NbGLR2.9b* were selected in the *Nb* WT. T3 homozygotes without *Cas9* were used. Stable transformants of *nrg1-5* expressing native promoter-driven NRG1-4xmyc WT or NRG1-4xmyc variants were generated by *Agrobacterium*-mediated transformation (71). Transgenic *Nb* WT harboring *CaMV35S:GCaMP3* (58) was crossed with *nrg1-5* and *glr2.9ab*_ln1. Kanamycin served as a selection marker. T3-generation homozygous mutant lines with the *CaMV35S:GCaMP3* transgene were used for cytosolic calcium measurements. *Nb* plants were grown in 16 h photoperiod-controlled environment rooms at 22 to 24 °C and 65% relative humidity for 4 to 5 wk.

### Construct Generation by Golden Gate Cloning.

Constructs for expressing NRG1 WT and variants were prepared using the Golden Gate MolClo kit (72).

The promoter+3’UTR of *Nb NRG1* (830 bp) was cloned into level 0 plasmid pICH41295. *NRG1* CDS was cloned into level 0 plasmid pAGM1287. *NRG1^D16A/D24A^* and *NRG1^G22A6/K227A^* were generated using the QuikChange II Site-Directed mutagenesis (SDM) protocol (#200555, Agilent). To obtain level 1 NRG1 constructs, level 0 constructs of NRG1/variants were combined with pICH41295 containing *NRG1pro*, 4xmyc (pICSL50010), CaMV *35S* terminator (pICH41414), and backbone pICH47732. The promoter+3’UTR of *Nb GLR2.9a* (1,916 bp) and *GLR2.9b* (1,579 bp) were cloned into level 0 plasmid pICH41295. Genomic *GLR2.9a* and *GLR2.9b* were separately cloned into level 0 plasmid pAGM1287. Expression level 1 constructs for *GLR2.9a* (*35S:gGLR2.9a-mCherry* and *pGLR2.9a:gGLR2.9a-mCherry*) and *GLR2.9b* (*35S:gGLR2.9b-GFP* and *pGLR2.9b:gGLR2.9b-GFP*) were cloned using the above strategy except the modules were cauliflower mosaic virus (CaMV) 35S promoter (pICH51288), *GLR2.9apro* or *GLR2.9bpro,* g*GLR2.9a* or g*GLR2.9b,* GFP tag *(*pICSL50008*)* or mCherry tag *(*pICSL50004*)*. Level 1 Golden Gate expression constructs were transformed via electroporation into *Rhizobium radiobacter* (hereafter *Agrobacterium tumefaciens* or Agrobacteria) strain GV3101 pMP90RK or pMP90 for transient expression in *Nb* or stable transformation of *Nb*. Primers for cloning and SDM are listed in *SI Appendix*, Table S1.

### *Agrobacterium*-Mediated Transient Expression in *N. benthamiana*.

*A. tumefaciens* strains were grown on YEB plates containing appropriate antibiotics and incubated 1 d at 28 °C. Cells were collected from plates and resuspended in induction buffer (10 mM MES pH 5.3, 10 mM MgCl_2_,150 nM acetosyringone) at OD_600_ = 0.2. Silencing suppressors p19 and CMV2b (OD600 = 0.1) were coinfiltrated into leaves of 4- to 5-wk-old *Nb* leaves with a 1 mL needleless syringe. In cell death assays, leaves were spot-infiltrated and evaluated for macroscopic tissue collapse 2 d post infiltration. Leaf discs (7 mm) were punched out of infiltrated zones at various time points for conductivity (ion leakage) measurements and frozen in liquid nitrogen for western blot analysis.

### *Pf*0-1 Infiltration into *N. benthamiana*.

Leaves of *Nb* were infiltrated with type III secretion system-equipped *Pseudomonas fluorescens* 0-1 (*Pf*0-1) strains resuspended at OD_600_ = 0.3 in 10 mM MgCl_2_. Mock infiltration used 10 mM MgCl_2_ only. Leaves for protein assays and RNA-seq were harvested at 6 hpi, flash-frozen in liquid nitrogen and stored at −80 °C. Leaves were visualized for macroscopic tissue-collapse and cell death measurement 1 d after the *Pf*0-1 strains infiltration.

### Cell Death Conductivity Assays.

*A. tumefaciens* or *Pf*0-1 (45) infiltrated *Nb* plants were kept at 22 °C with a 16-h light period. Eight 7-mm leaf discs were taken at specified timepoints, washed in 20 mL of milliQ water for 60 min, transferred to a 48-well plate with 0.5 mL milliQ in each well, and incubated at room temperature under light. Ion leakage (conductivity) was measured at 0 h and 24 h with a conductometer, Horiba Twin ModelB-173. For statistical analysis, results of measurements for individual wells (each leaf disc in one well represents a technical replicate) were combined from independent experiments (biological replicates).

### In Planta Bacterial Growth Assays.

*Xe* 85-10 and *Xe*Δ*XopQ* were resuspended in 10 mM MgCl_2_ at OD_600_ = 0.001 and syringe-infiltrated into *Nb* leaves. Plants were kept in a long-day chamber (16 h light/ 8 h dark at 24 °C/22 °C). Bacteria were isolated at 0 dpi and 6 dpi (each with four 7-mm leaf discs representing four technical replicates), and dilutions were dropped onto NYGA plates supplemented with rifampicin 100 mg/L and streptomycin 150 mg/L. In statistical analysis of *Xe* and *Xe*Δ*XopQ* titers at 6 dpi, results from independent experiments (biological replicates) were combined.

### Nuclear/Cytoplasm Fractionation.

Leaf tissue (1.5 g fw) was homogenized in 3 mL Honda buffer (2.5% Ficoll 400, 5% Dextran T40, 0.4 M sucrose, 25 mM Tris-HCl, pH7.4, 10 mM MgCl_2_ (with 5 mM DTT, 1× protease inhibitor cocktail added before use). The lysate was filtered through a 62 µm nylon mesh, by centrifuging for 3 min at 1,000 rpm. After lysate collection, all steps were conducted at 4 °C or on ice. Triton X-100 was added to the lysate (final concentration of 0.5%) and mixed slowly followed by 15 min incubation on ice [− the total fraction aliquot (Input) was taken here]. The lysate was then centrifuged at 1,500 g for 5 min, the pellet retained, and the supernatant centrifuged at 13,000 g for 15 min, and a 500 mL aliquot taken as PM/C sample. The pellet was resuspended in 2.5 mL Honda buffer with 0.1% Triton X-100 and centrifuged again at 1,500 g for 5 min. After discarding the supernatant, the pellet was resuspended in 2.5 mL Honda buffer without Triton X-100. This suspension was transferred to two 1.5 mL Eppendorf tubes and centrifuged at 100 g for 5 min to remove remaining debris. The suspension was then centrifuged at 2,000 g for 5 min to pellet nuclei. The pellet was resuspended in 300 µL buffer G (1.7 M sucrose, 10 mM Tris-HCl pH 8.0, 0.15% Triton X-100, 2 mM MgCl2, 5 mM DTT, 1× protease inhibitor cocktail) and layered onto 300 µL buffer G in a new 1.5 mL Eppendorf tube followed by centrifugation at 16,000 g for 1 h. The supernatant was removed and the pellet resuspended in 100 µL Honda buffer and 100 µL 2× SDS loading buffer (N sample). Loading equal volumes of both PM/C and N fractions (10 µL) on a gel resulted in a 16-fold overrepresentation of the nuclear-enriched vs. PM/C sample.

### Western Blot Analysis.

Two 9 mm leaf discs were frozen in liquid nitrogen and homogenized at 30 Hz for 60 s with 1 mm metal beads in a TissueLyser II (Qiagen, Netherlands). Total protein was extracted from the fine powder in 100 µL 2× Laemmli buffer (125 mM Tris-HCl pH6.8, 4% [v/v] SDS, 20% [v/v] glycerol, 200 mM DTT, 0.02% [w/v] bromophenol blue). Samples were boiled at 95 °C for 7 min and separated by SDS-PAGE (Any kD™ Mini-PROTEAN® TGX™ Precast Protein Gels, BioRad). Antibodies used for immunoblotting were α-myc (71D10, Cell Signaling), α-mCherry (E5D8F, Cell Signaling), α-GFP (11814460001, Roche), α-PEPC (Rockland 100-4163), α-H3 (ab1791, Abcam), HRP-conjugated antibodies (Anti-mouse IgG-HRP: sc2005, Santa Cruz Biotechnology; Anti-rabbit IgG-HRP: 31460, Invitrogen; Anti-rat IgG-HRP: sc2006, Santa Cruz Biotechnology). The dilution of primary and secondary antibodies was 1:5,000 (2% nonfat dry milk in TBST), except for α-PEPC, which was used at a dilution of 1:1,000. Membranes were developed using ClarityWestern ECL Substrate (Bio-Rad, USA) for low sensitivity detection and Clarity Max Western ECL Substrate (Bio-Rad, USA) for high sensitivity detection in a ChemiDoc (Bio-Rad, USA) device. For loading control, membranes were stained with Ponceau S (09276-6X1EA-F, Sigma-Aldrich).

### Confocal Microscopy and Imaging.

*A. tumefaciens* strains carrying *35S:gGLR2.9b-GFP or 35S:gGLR2.9a-mCherry* were syringe-infiltrated into leaves of *Nb glr2.9ab* for live cell imaging at 2 dpi. Agrobacteria carrying *pGLR2.9b:gGLR2.9b-GFP* or *pGLR2.9a:gGLR2.9a-mCherry* were syringe-infiltrated into leaves of *Nb glr2.9ab* for live cell imaging. At 2 dpi, the leaf zone was infiltrated with OD_600_ = 0.3 *Pf*0-1 *XopQ* or *Pf0*-1 EV in 10 mM MgCl_2_, or 10 mM MgCl_2_ (mock). At 6 hpi, leaf discs were harvested for confocal microscopy imaging on a Zeiss LSM780 confocal laser scanning microscope. Wavelengths of 488 nm and 510 nm were used for excitation and detection of GFP. Wavelengths of 580 nm and 610 nm were used for excitation and detection of mCherry. Objectives used were 20× (0.8 NA, water). Confocal images were compiled using Fiji 1.53c. Two 9 mm leaf discs per sample were collected for western blot analysis.

### ROS Burst Assay.

Leaf discs (~0.5 cm) were collected from 4-wk-old *Nb* leaves and incubated overnight in 200 μL sterile water in a 96-well plate. The discs were treated with a reaction solution containing 100 μM luminol and 1 mg/mL horseradish peroxidase supplemented with or without 1 μM flg22 peptide. Luminescence was measured using the GLOMAX96 microplate luminometer (Promega, Madison, WI).

### Cytoplasmic Calcium Measurements in *Nb* Leaves.

GCaMP3 fluorescence signals were recorded in a temperature-controlled (22 °C) dark room using a motorized fluorescence stereo microscope Nikon SMZ25 with 0.5 × objective lens (SHR Plan Apo, WD:71, Nikon) and a high-resolution Nikon DS-Ri2 camera. A Nikon GFP-BP filter cube (EX: 470/40 and EM: 525/50) was used for excitation. GCaMP3 signals were captured every 5 min and analyzed with NIS-Elements Advanced Research microscope imaging software. For time-lapse measurements, 3-wk-old *Nb* were used. Immediately after the infiltration of mock, *Pf*0-1 EV, or *Pf*0-1 *XopQ*, detached leaves were placed on black cardboard over wet tissue and sealed in a plastic Petri dish. Measurements started after setting the exposure time to 7 s. Fluorescence was visualized using a “Plot Z-axis profile.” Fiji 1.53c was used for GCaMP3 signal analysis (59). Formula (F_t_−F_0_)/F_0_ were used for GCaMP3 fluorescence signal analysis. F_t_: signal intensity at time t. F_0_: baseline signal intensity measured at t = 0.

### Phylogenetic Tree Construction.

A list and amino acid sequences of *Nb* GLRs was obtained from Sol Genomics Network annotation file Niben101_annotation.functional.txt in the FTP database (https://solgenomics.net/). Sequences of *A. thaliana* GLRs were obtained from The *Arabidopsis* Information Resource (https://www.arabidopsis.org/). Amino acid sequences were aligned by MAFFT (v7.505) with the auto parameter, using the L-INS-i strategy. From the aligned files, the tree files were generated by IQ-TREE2 (2.2.2.1) (73, 74) AUTO parameter with the option SH-alrt and -B to bootstrap 1,000 times (75) for branch support. The phylogenetic tree was plotted with iTOL from the generated tree file.

### RNA Extraction, Reverse Transcription, and qRT-PCR.

Two 9 mm leaf discs were frozen in liquid nitrogen and homogenized at 30 Hz for 60 s with 1 mm metal beads in a TissueLyser II (Qiagen, Netherlands). RNA was extracted using a my-Budget Plant RNA Kit (Bio-Budget Technologies, Germany) according to instructions. Extracted RNA was treated with DNase I to remove residual DNA. For reverse transcription, 1 µg total RNA was treated according to RevertAid First Strand cDNA Synthesis Kit instructions (Thermo Fisher Scientific, USA). One µL 12.5 ng/µL cDNA was mixed with 0.5 µL 10 mM forward primer, 0.5 µL 10 mM reverse primer and 5 µL SYBR Green (Bio-Rad iQ SYBR® Green Supermix) for qPCRs. Quantitative RT-PCR assays were performed using a CFX Connect™ Real-Time System Thermal cycler (Bio-Rad). Gene expression levels were quantified with the 2^−ΔΔCq^ method and *NbACT2* as reference house-keeping gene.

### RNA-seq Analysis.

Mock, *Pf*0-1 EV, and *Pf*0-1 *XopQ* (OD_600_ = 0.3) were inoculated into leaves of *Nb* WT, *nrg1-5*, *epss,* and *epssna*. Nine 8 mm leaf discs were collected from three leaves of three plants per treatment to constitute one biological replicate. Three biological replicates were collected and frozen in liquid nitrogen 6 h after inoculation. Total RNA was extracted using TRIzol (15596018, Invitrogen). RNA purification was performed using a ReliaPrep™ RNA Miniprep System (Promega Z6112) following the manufacturer’s instructions. Eluted RNA was quantified on a NanoDrop spectrophotometer. Each sample was sent to Novogene for library preparation and RNA sequencing. RNA sequencing was performed using a Novaseq 6000 Illumina platform with V1.5 reagent. Raw FASTQ files were quality-controlled by the FastQC tool (v0.11.9). Quality controlled reads from raw FASTQ files were aligned to the *N. benthamiana* reference genome version v1.0.1 (https://solgenomics.net/). DEG were identified by using R package DESeq2 (v1.38.0). Only genes with DESeq2 normalized counts ≥ 10 in at least 3 out of 12 samples were analyzed to reduce the number of false positive DEG. Threshold cut-offs for DEG were |log2 fold change| ≥ 1 (fold change of 2) and a false discovery rate corrected *P*-value < 0.05.

### GO Term Enrichment Analysis.

Functional annotations of genes including Gene Ontology terms (GO terms) and orthologs in other species were obtained from the Phytozome database (https://phytozome-next.jgi.doe.gov/). Shiny GO (76) was used for GO term enrichment analyses of the DEG clusters, with an enrichment threshold of *P*-value ≤ 0.05.

### *Nb* GLR2.9a and GLR2.9b Protein Structure Modelling.

*Nb*GLR2.9a and *Nb*GLR2.9b structure models were prepared with SWISS-MODEL based on the cryo-EM structure of *At*GLR3.4 (PDB: 7LZH). An *Nb*NRG1 structure model was prepared with SWISS-MODEL based on the *At*ZAR1 pentamer (PDB: 6J5T). Visualization of the structures was performed in Chimera (version 1.17.1).

### Software Used for Visualization and Statistical Analysis of Data.

Statistical analyses were performed in R (4.2.3). Data were first checked for normality of residuals distribution and homogeneity of variance by examining the plots and Shapiro–Wilk and Levene tests (*P* > 0.05). If both conditions were met, ANOVA was followed by a Tukey honestly significant difference test (α = 0.05). Otherwise, The Nemenyi test with Bonferroni correction for multiple testing was applied (α = 0.05). Heatmaps were generated with the ComplexHeatmap package. Venn diagrams were plotted by the VennDiagram (1.7.3) package. PCA was performed with the vegan (2.6.4) package. Boxplots and Ribbon plots were generated with the ggplot2 (3.4.2) package. All packages were installed in R (4.2.3).

## Supplementary Material

Appendix 01 (PDF)

Dataset S01 (XLSX)

Dataset S02 (XLSX)

## Data Availability

RNA-seq data generated in this study are deposited at the European Nucleotide Archive database (submissions 7-9 April, 2025) under accession code: PRJEB88173 (https://www.ebi.ac.uk/ena/browser/view/PRJEB88173) (77). Materials generated in the paper will be made available to academic researchers under an MTA. All other data are included in the manuscript and/or supporting information.
