# Supplementary material for: Coordinated actions of NLR-assembled and glutamate receptor–like calcium channels in plant effector-triggered immunity
Source: Proc Natl Acad Sci U S A. 2025 Aug 22;122(35):e2508018122. doi: 10.1073/pnas.2508018122 (PMC12415192; doi:10.1073/pnas.2508018122)
Supplement: Supplementary file 1 — Appendix 01 (PDF) [file pnas.2508018122.sapp.pdf]

Supporting Information for

Coordinated actions of NLR-assembled and glutamate receptor-like calcium channels in plant effector-triggered immunity

Junli Wang[a,1,*], Xinhua Sun[a,b,1], Fei Xiong[a], Dmitry Lapin[a,c], Tak Lee[a], Sergio Martin-Ramirez[a,d], Anna Prakken[e], Qiaochu Shen[e,f], Jaqueline Bautor[a], Takaki Maekawa[e,g], Jane E. Parker[a,g,*]

[a]Department of Plant-Microbe Interactions, Max Planck Institute for Plant Breeding Research, Cologne 50829, Germany

[b]current address: Chongqing Key Laboratory of Plant Disease Biology, College of Plant Protection, Southwest University, Chongqing 400715, People's Republic of China

[c]current address: Department of Biology, Translational Plant Biology, Utrecht University, 3584 CH Utrecht, the Netherlands

[d]current address: Laboratory of Biochemistry, Wageningen University & Research, the Netherlands

[e]Institute for Plant Sciences, University of Cologne, Cologne 50674, NRW, Germany

[f]Department of Molecular, Cellular & Developmental Biology, University of Michigan, Ann Arbor, MI 48109, USA

[g]Cluster of Excellence on Plant Sciences, Max Planck Institute for Plant Breeding Research, Cologne 50829, NRW, Germany

* To whom correspondence should be addressed. Email: parker@mpipz.mpg.de; jwang@mpipz.mpg.de

[1] These authors contributed equally

This PDF file includes:

- Figures S1 to S10
- Table S1
- Legends for Datasets S1 to S2
- Detailed Material and Methods

Other supporting materials for this manuscript include the following:

- Datasets S1 to S2

Supplementary Figures

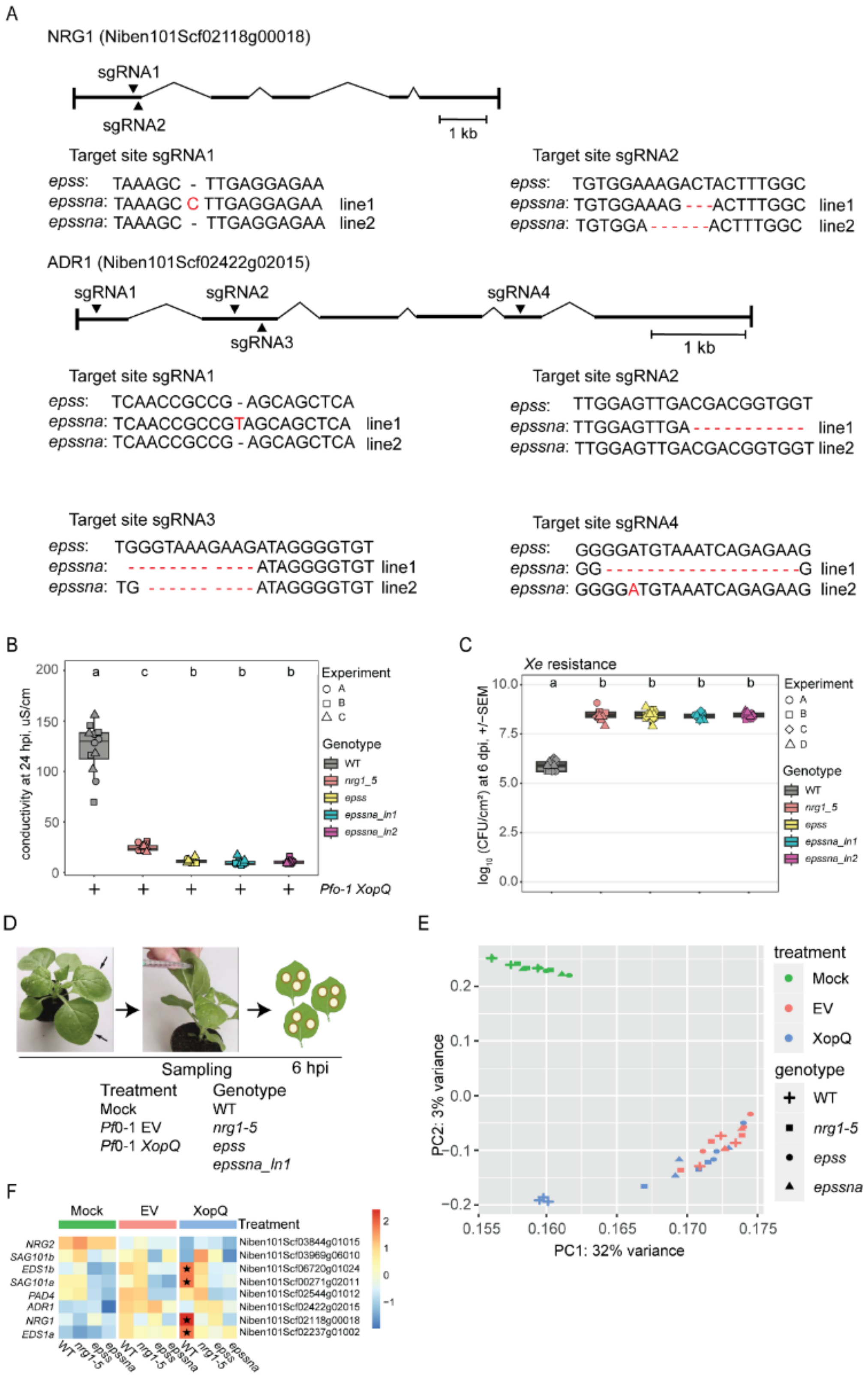

Fig. S1. Analysis of XopQ-triggered cell death/resistance phenotypes and transcriptional changes and their dependence on the EDS1-SAG101-NRG1 node in *N. benthamiana*. **(*A*)** Molecular details on *epssna* CRISPR-Cas9 mutants generated in this study. *NRG1* and *ADR1* loci were simultaneously targeted for genome editing using sgRNAs in an *Nb epss* background. The locus with positions of target sites are shown as a gene schematic and nucleotide sequence. The predicted cut site for each

guide is indicated with an arrow and the inserted/deleted DNA bases are in red. **(*B*)** Conductivity measurements of Roq1-mediated cell death in *Nb* WT*, nrg1-5, epss*, *epssna*_ln1 and *epssna*_ln2 at 1 dpi using *Pf*0-1 *XopQ* (n = 12 from three independent experiments). Lowercase letters indicate significant differences measured by one-way ANOVA followed by post hoc test (α = 0.05). **(*C*)** *Xe* growth assay in *Nb* WT, *nrg1-5, epss*, *epssna*_ln1 and *epssna*_ln2 leaves at 6 dpi with *Xe* (n = 16 from four independent experiments). Lowercase letters indicate significant differences measured by one-way ANOVA followed by post hoc test (α = 0.05). (*D*) Experimental design and sampling for RNA-seq analysis. *Nb* WT*, nrg1-5*, *epss* and *epssna*_ln1 were infiltrated with $MgCl_2$ (mock), *Pf*0-1 EV or *Pf*0-1 *XopQ*. Nine leaf discs from 3 plants were collected at 6 hpi of each treatment as one biological replicate. **(*E*)** PCA analysis of all RNA-seq normalized counts. (*F*) A heatmap representation of EPA and EDS1-SAG101-NRG1 node transcript changes in different *Nb* genotypes, as indicated, at 6 h post $MgCl_2$ (mock), *Pf*0-1 EV (EV) or *Pf*0-1 *XopQ* (XopQ) infiltration. Transcript values relative to mean normalized counts of all RNA-seq samples are shown on a scale of red (higher than the mean), yellow (close to the mean) and blue (lower than the mean). Stars indicate differentially expressed transcripts.

A

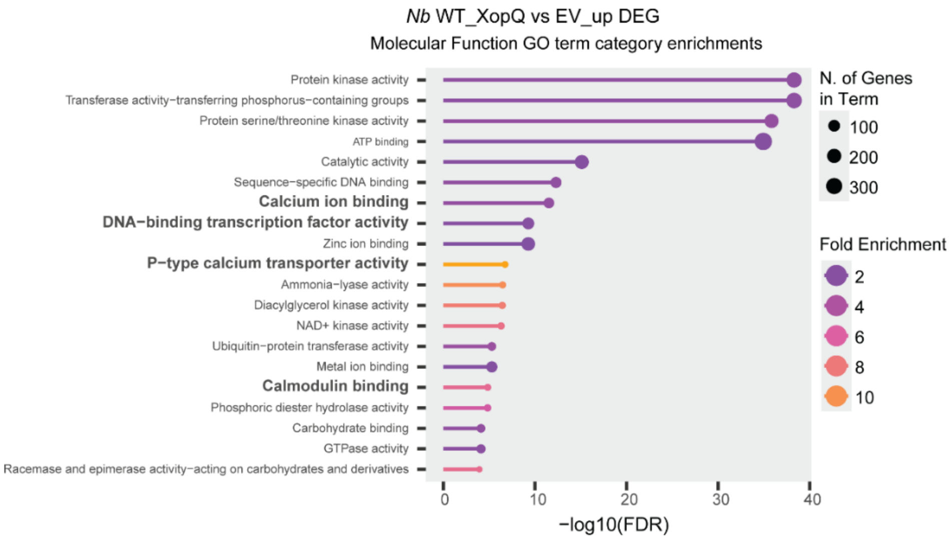

B

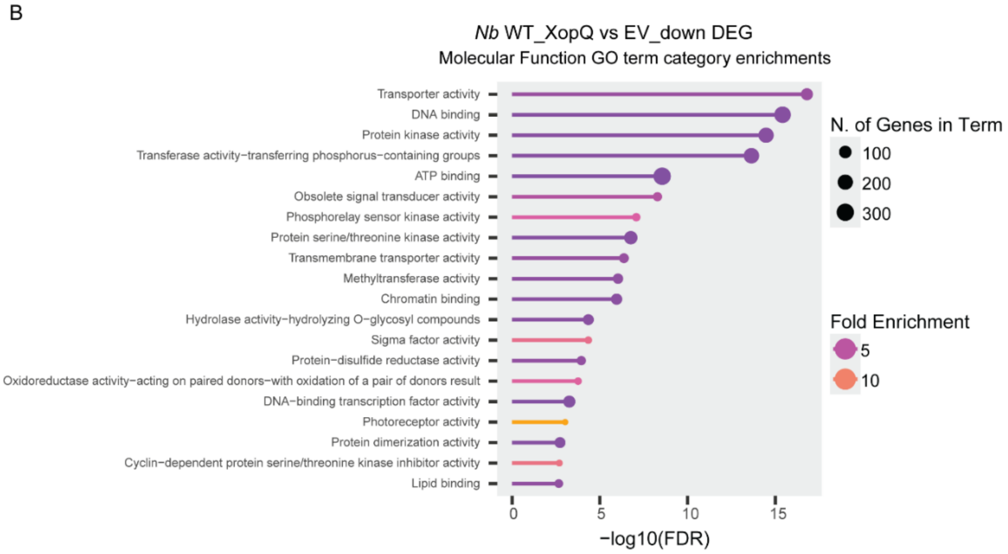

Fig. S2. Top 20 GO terms (Molecular Function) enriched in ETI-specific up- and down-DEG in *Nb* WT (XopQ vs EV). **(*A*)** Selected GO terms enriched in the set of 3876 up-DEG in *Nb* WT (XopQ vs EV). (***B*)** Selected GO terms enriched in the set of 4187 down-DEG in *Nb* WT (XopQ vs EV). Size of the dots reflect numbers of DEG annotated in that GO term. Dots colour indicates fold enrichment significance. Dataset S2 lists all enriched ETI-specific GO terms in *Nb* WT (XopQ vs EV).

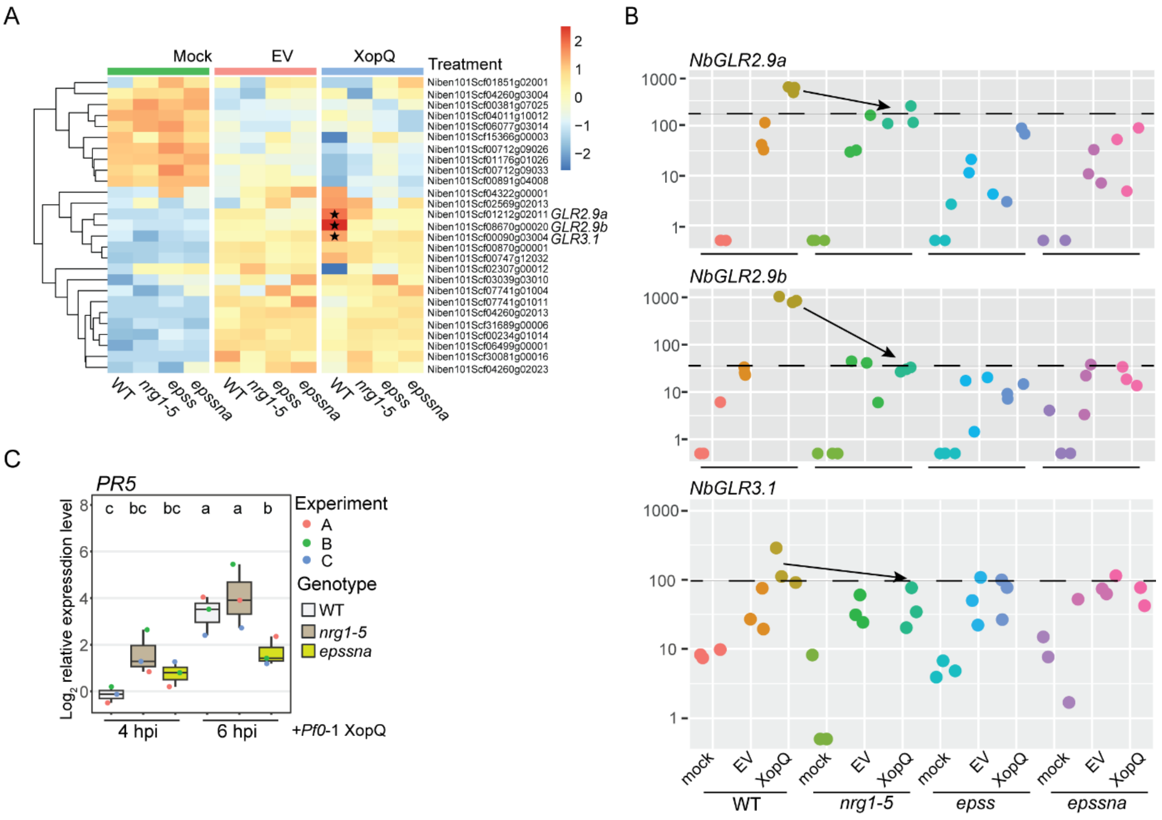

Fig. S3. RNA-seq analysis of XopQ-triggered changes in *Nb* WT*, nrg1-5*, *epss* and *epssna*. **(*A*)** A heatmap representation of *GLR* transcript changes in different *Nb* genotypes, as indicated, at 6 h post $MgCl_2$ (mock), Pf0-1 EV (EV) or *Pf*0-1 *XopQ* (XopQ) infiltration. Transcript values relative to mean normalized counts of all RNA-seq samples are shown on a scale of red (higher than the mean), yellow (close to the mean) and blue (lower than the mean). Stars indicate differentially expressed transcripts for genes of interest. **(*B*)** Comparison of the number of normalized counts of *Nb GLR2.9a*, *GLR2.9b* and *GLR3.1* in different *Nb* genotypes, as indicated, at 6 h post $MgCl_2$ (mock), *Pf*0-1 EV (EV) or *Pf*0-1 *XopQ* (XopQ) infiltration. **(*C*)** qRT-PCR analysis of *NbPR5* expression in *Nb* WT, *nrg1-5* and *epssna* at 4 h and 6 h post infiltration with *Pf*0-1 *XopQ*. Samples are normalized to *Nb* WT at 4hpi with *Pf*0-1 EV (PTI). Data were normalized using Log_2 due to differences in the induced expression of the tested genes. Data are from three biological replicates. The Nemenyi test with Bonferroni correction for multiple testing was applied ($\alpha = 0.05$). Different lowercase letters indicate significant difference.

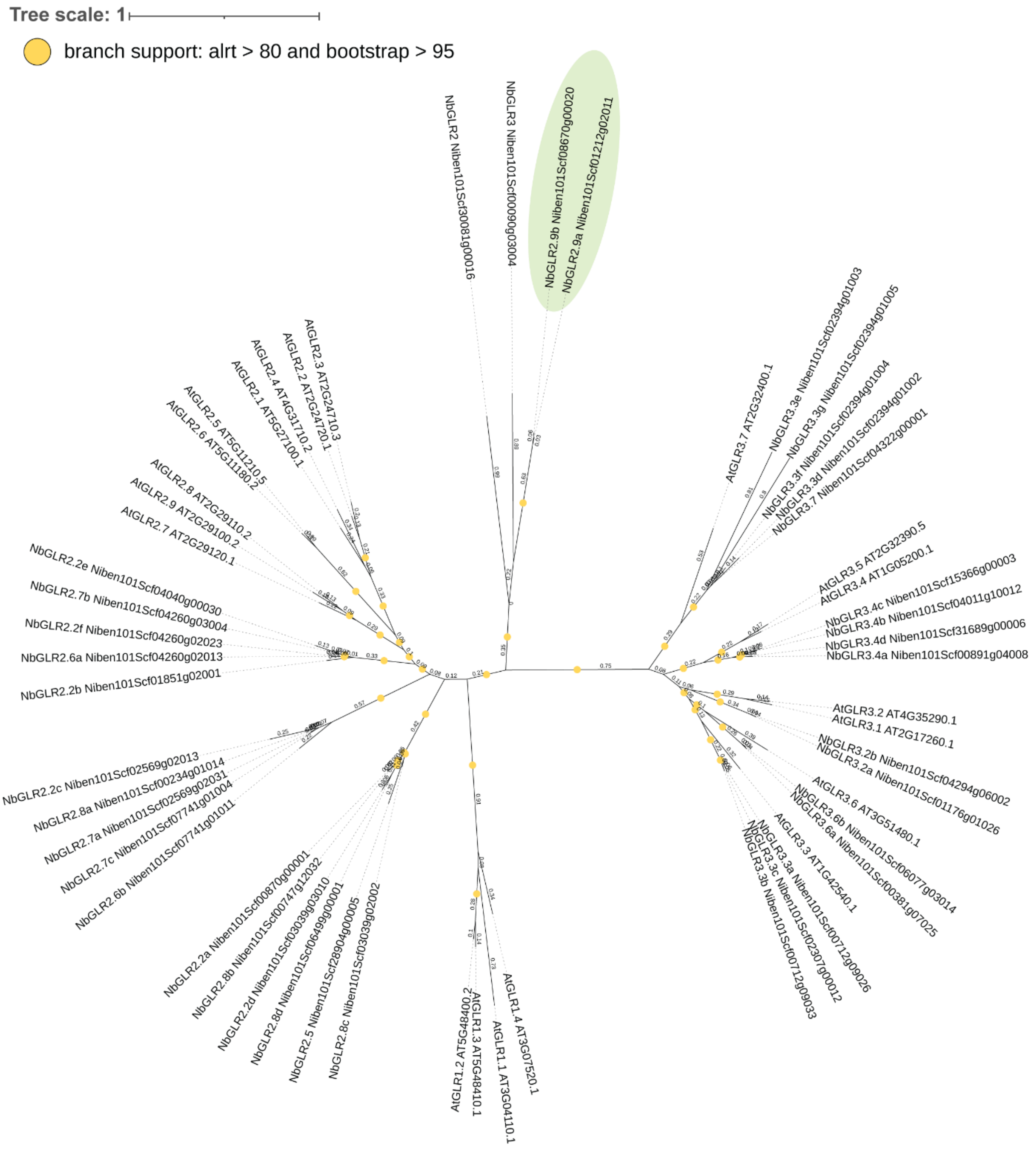

Fig. S4. An unrooted phylogenetic tree based on full-length *Nb*GLR and *At*GLR sequences. The phylogenetic tree indicates sequence relationships between GLR family members in *N. benthamiana* and *Arabidopsis thaliana*. Sequence alignment and tree construction were performed using MAFFT and IQ-TREE2, respectively. Branch lengths represent evolutionary distance, and bootstrap values shown at major nodes indicate statistical confidence of the inferred relationships. Yellow circles denote branch support values alrt > 80 and bootstrapping > 95.

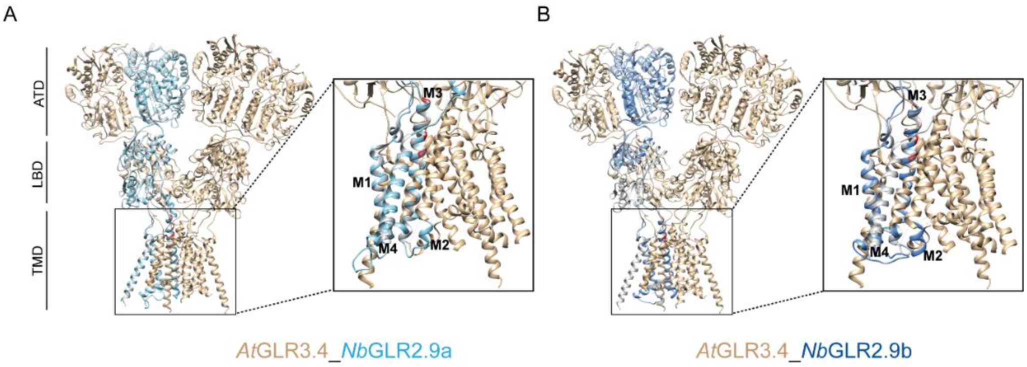

C

```
                                            ATD-LBD linker     start_LBD_S1
AtGLR3.4_aa_sequence      STANQRLKGIIYPGEVTKPPRGWVFPNNGKPLRIGVPNRVSYTDYVSKDK----NPPGVR 518
GLR2.9a_aa_sequence       GSHTMNSSEVKWPGELNRVPKGWAMPTDSKPLIIGVPGRTSFEKFVKVEKVANSNERKYS 481
GLR2.9b_aa_sequence       GSHTMKSNEVKWPGELNRVPKGWAMPTDSKPLFIGVPGRTSFEKFVKVKKVGNSNERKYS 448
                          .:  .  .  .  :  :***:.:  *:**.:*.:.***  ****.*.*:  .:*.  .*     *

AtGLR3.4_aa_sequence      GYCIDVFEAAIELLPYPVPRTYILYGDGKRNPSYDNLVNEVVADNFDVAVGDITIVTNRT 578
GLR2.9a_aa_sequence       GFCIDLFKEVLKILEQNYTLP---YEFEPYDGSYPDLVQHVINRSYDAIVGDMTILAERI 538
GLR2.9b_aa_sequence       GFCIDLFKEVLKSLEQNYTLP---YEFEPYDGSYPDLVEQLINRRYDAIVGDMTILAERI 505
                          *:***:*:  .::  *          *      :  **  :**:.::    :*.  ***:**:::*

                                    end_LBD_S1                        M1
AtGLR3.4_aa_sequence      RYVDFTQPFIESGLVVVAPVKE-AKSSPWSFLKPFTIEMWAVTGGFFLFVGAMVWILEHR 637
GLR2.9a_aa_sequence       KYVEFTQPFAESGLTMVVPVKFDGSKKAWMFLKPFTISMWIVTGSILVYTMLVVWFMEHQ 598
GLR2.9b_aa_sequence       KYVEFTQPFAESGLTMVVPVKFDGPKKAWMFLKRFTISMWIVTGSILVYTMLVVWFMEHQ 565
                          :**:***** ****.:*.***    .  ..  *  ***  ***.**  ***.::::.   :**::**:

                                    M2                          M3
AtGLR3.4_aa_sequence      FNQEFRGPPRRQLITIFWFSFSTMFFSHRENTVSSLGRFVLIIWLFVVLIINSSYTASLT 697
GLR2.9a_aa_sequence       SNPEFRGRWKDQLGTAMWFTFSSLFFAHREKIKSNYTKIVVVVWLFLVFVLTSSYTASLT 658
GLR2.9b_aa_sequence       SNPEFRGRWRDQLGTAMWFTFSSLFFAHRENIKSNCTKIVVVVWLFLVFVLTSSYTASLT 625
                           *  ****   :  **  *  :**:**::**:***:   *.   ::*:::***:*:::.********

                                   start_LBD_S2
AtGLR3.4_aa_sequence      SILTIRQLTSRIEGIDSLVTSNEPIGVQDGTFARNYLINELNILPSRIVPLKDEEQYLSA 757
GLR2.9a_aa_sequence       SMLTVPRLEPSVKDINWIKRTNTTVGCDGDSFVKDYLRQVLELQNI--KNISNQEDYPKE 716
GLR2.9b_aa_sequence       SMLTVPRLEPSVKDIN-------------------------------------------- 641
                          *:**:  :*    ::.*:

AtGLR3.4_aa_sequence      LQRGPNAGGVAAIVDELPYIEVLLTNSNCKFRTVGQEFTRTGWGFAFQRDSPLAVDMSTA 817
GLR2.9a_aa_sequence       F----ESGNIKAAFLEIPYQKVFLREHCNQYVVAGPNYRFGGLAFVFQKGSPLARDVSEA 772
GLR2.9b_aa_sequence       ------------------------------------------------------------ 641

                                      end_LBD_S2                    M4
AtGLR3.4_aa_sequence      ILQLSEEGELEKIHRKWLNYKHECSMQISNSEDSQLSLKSFWGLFLICGITCFMALTVFF 877
GLR2.9a_aa_sequence       ILTLTQDGTLNSLEERWFALSRNCDNVDASNETESLTLGSFWGLYLVSGATSTVCLLFFI 832
GLR2.9b_aa_sequence       ------------------------------------------------------------ 641

AtGLR3.4_aa_sequence      WRVFWQYQRLLPESADEERAGEVSEPSRSGRGSRAPSFKELIKVVDK--REAEIKEIL-- 933
GLR2.9a_aa_sequence       WQLCRKS----PQQSRVYHEN-IVLPSTDQ--SFWTKTAALIRYYQNDNPAASLRRLTSG 885
GLR2.9b_aa_sequence       ------------------------------------------------------------ 641

AtGLR3.4_aa_sequence      --------KQKSSKKL------KSTQSAAGTSQSQHG-----EIT--  959
GLR2.9a_aa_sequence       RAAGLGVEERGSSPKWHLVSPSDAAQIFDGSSQHPQSSLEMGNLRSD  932
GLR2.9b_aa_sequence       -----------------------------------------------  641
```

Fig. S5. Remodelled structures of *Nb*GLR2.9a and *Nb*GLR2.9b. Structures of GLR2.9a **(*A*)** and GLR2.9b **(*B*)** modelled on tetrameric *At*GLR3.4 (PDB: 7LZH) using SWISS-MODEL. The *At*GLR3.4 tetramer is colored tan. The *At*GLR3.4 monomer is colored dark grey. GLR2.9a is colored sky blue and GLR2.9b dark blue. The boxes show magnified TMDs of GLR2.9a (*A* right) and GLR2.9b (*B* right) with gate forming amino acids highlighted in red. **(*C*)** Amino acid sequence alignment of *At*GLR3.4 encompassing LBD (S1 and S2 sequences in red) and TMD (M1-M4 sequences in pink) regions with *Nb*GLR2.9a and *Nb*GLR2.9b. Key residues in LBD S1/S2 contributing to ligand binding are indicated with black (conservative) or brown (non-conservative) arrowheads. Gate-forming residues within the M3 region (aa 671 to 708) are indicated with blue arrowheads. Conserved residues between the three sequences are in bold.

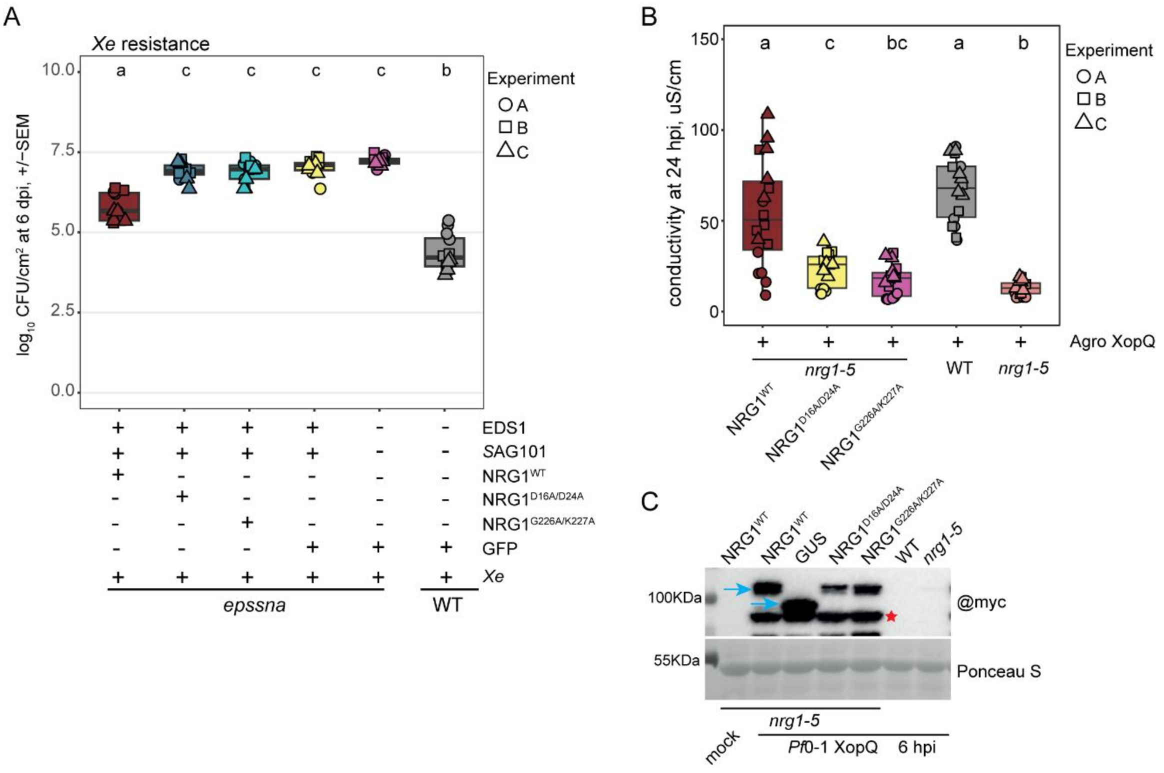

Fig. S6. NRG1^{WT} but not NRG1$^{\alpha 1\text{-helix}}$ or NRG1$^{\text{P-loop}}$ variants rescue *nrg1-5* immunity defects. **(*A*)** *Xe* growth assay in *Nb epssna* leaves. 4xmyc-tagged NRG1^{WT}, NRG1$^{\mathrm{D16A/D24A}}$, NRG1$^{\mathrm{G226A/\ K227A}}$ or GFP were co-infiltrated with *Nb*EDS1, *Nb*SAG101 and *Xe* into *Nb epssna*. GFP was co-infiltrated with *Xe* into *Nb* WT as a positive control. *Xe* bacteria were counted in leaves at 6 dpi for pathogen growth analysis (n = 12 from three independent experiments). Lowercase letters indicate significant differences by one-way ANOVA followed by post hoc test ($\alpha = 0.05$). **(*B*)** Conductivity measurements of XopQ-triggered cell death at 2 d after agroinfiltration of NRG1^{WT}, NRG1$^{\mathrm{D16A/D24A}}$, NRG1$^{\mathrm{G226A/K227A}}$ together with XopQ in *nrg1-5*. *Nb* WT and *nrg1-5* agroinfiltrated with XopQ served as positive and negative cell death control (n = 15 from three independent experiments). Lowercase letters indicate significant differences by one-way ANOVA followed by post hoc test ($\alpha = 0.05$). **(*C*)** Western blot analysis of NRG1-4xmyc protein. 4xmyc-tagged NRG1^{WT}, GUS, NRG1$^{\mathrm{D16A/D24A}}$ and NRG1$^{\mathrm{G226A/K227A}}$ stable transgenic lines were infiltrated with *Pf*0-1 *XopQ* and samples harvested at 6 hpi for Western blot analysis. NRG1^{WT} protein was undetectable under mock_$MgCl_2$ conditions. *Nb* WT and *nrg1-5* without treatment served as negative control. The red star indicates an unspecific band. Three independent blots showed similar results.

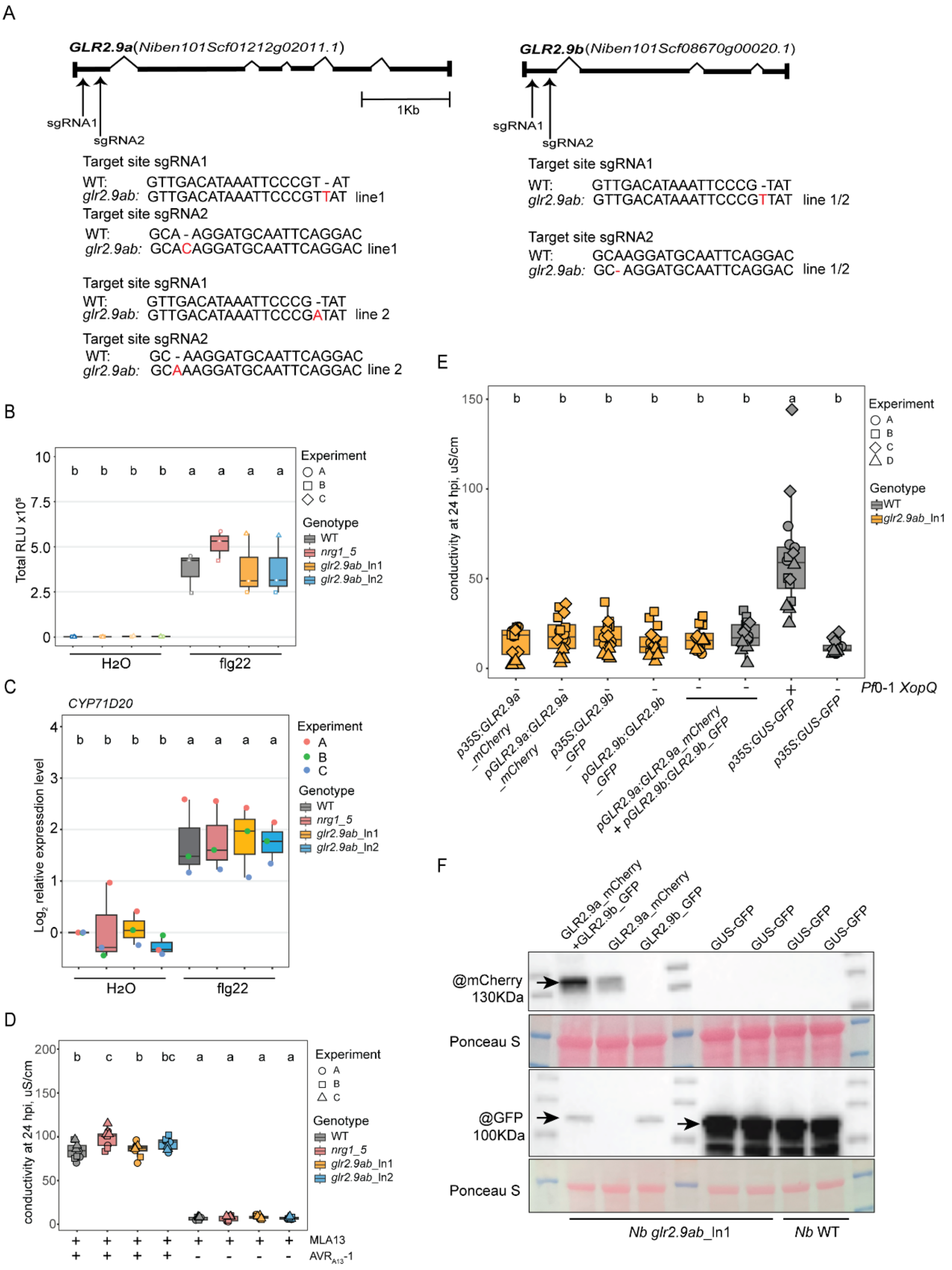

Fig. S7. GLR2.9a/GLR2.9b do not contribute to PTI and CNL-triggered cell death and transient expression of GLR2.9a-mCherry and/or GLR2.9b-GFP does not induce cell death in *Nb glr2.9ab*. **(*A*)** Molecular details of *glr2.9ab* CRISPR-Cas9 mutants generated in this study. *GLR2.9a* and *GLR2.9b* loci were simultaneously targeted for genome editing using sgRNAs in the *Nb* WT background. Each locus with positions of target sites is shown as a gene schematic and nucleotide sequence. The predicted cut site for each guide is indicated with arrows and inserted/delected DNA bases are in red. **(*B*)** Quantification of total flg22-induced ROS burst under curveshown in Fig. 4*D*. **(*C*)** GLR2.9a and GLR2.9b are not essential for flg22-induced expression of PTI defense-related gene *CYP71D20*. Leaves of *Nb* WT, *nrg1-5*, *glr2.9ab*_ln1 and *glr2.9ab*_ln2 were infiltrated with 1 µM flg22 and RNA extracted at 3 h. Samples are normalized to *Nb* WT at 3 hpi with H_2O. Data were normalized using

Log_2 due to differences in expression. Data are from three biological replicates. **(*D*)** Conductivity measurements of CNL MLA13 triggered cell death in leaves of the indicated lines at 24 h post agroinfiltration of MLA13 with or without effector AVR_{A13}-1 (n = 9 from three independent experiments). **(*E*)** Transiently expressed GLR2.9a-mCherry or GLR2.9b-GFP do not display cell death-inducing autoactivity. Constructs: *p35S:gGLR2.9a-mCherry*, *pGLR2.9a:gGLR2.9a-mCherry*, *p35S:gGLR2.9b-GFP*, *pGLR2.9b:gGLR2.9b-GFP* in *glr2.9ab*, *p35S:GUS-GFP* and *pGLR2.9a:gGLR2.9a-mCherry* with *pGLR2.9b:gGLR2.9b-GFP* in *Nb* WT or *glr2.9ab*_ln1 are shown. At 1 d post agroinfiltration, *Pf*0-1 *XopQ* was infiltrated into leaf sectors of *p35S:GUS-GFP* in WT and all samples were collected at 2 d for conductivity measurements (n = 12 from three independent experiments). **(*F*)** Western blot analysis of samples taken from experiment shown in Figure 5 *A*. Three independent blots gave similar results. (***B***-***E***) Genotypes with different letter codes are significantly different. The one-way ANOVA followed by post hoc test was applied ($\alpha = 0.05$).

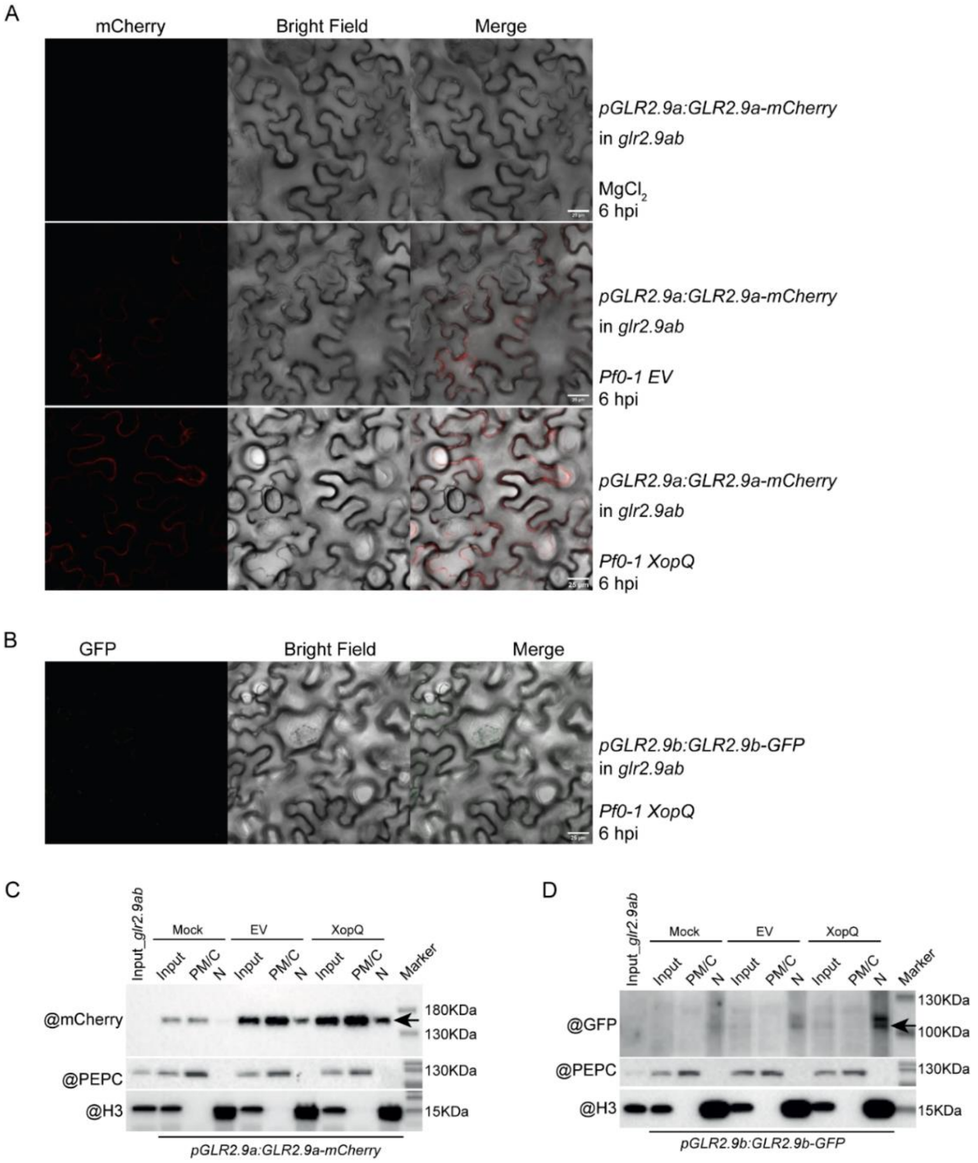

Fig. S8. Subcellular localization of *Nb*GLR2.9a and *Nb*GLR2.9b in *Nbglr2.9ab* transient immune response assays. **(*A*)** Native promoter-driven GLR2.9a-mCherry was detected at the plasma membrane/cytoplasm (PM/C) after *Pf*0-1 EV or *Pf*0-1 *XopQ* infiltration. At 2 d post agroinfiltration of the construct, mock_$MgCl_2$, *Pf*0-1 EV or *Pf*0-1 *XopQ* were infiltrated into the same leaf sectors. Samples were imaged at 6 hpi by confocal microscopy and analysed in three independent experiments (four cells imaged/experiment). Representative images of mCherry (red) signals are shown. Scale bar = 25 µm. **(*B*)** Native promoter-driven GLR2.9b-GFP was not detected using confocal microscopy in *Nb glr2.9ab*_ln1 leaves after *Pf*0-1 *XopQ* infiltration. At 2 d post agroinfiltration of the construct, *Pf*0-1 *XopQ* was infiltrated into the same leaf sectors. Samples were imaged at 6 hpi by confocal microscopy and analysed in three independent experiments (four cells imaged/experiment). Representative images of GFP (green) signals are shown. Scale bar = 25 µm. **(*C - D*)** Plasma membrane/cytoplasm (PM/C) and nuclear enriched (N) fractions of samples from the experiment shown in Fig. S8 *A - B*. Agrobacteria carrying *pGLR2.9a:gGLR2.9a-mCherry* or *pGLR2.9b:gGLR2.9b-GFP* constructs were infiltrated into *Nb glr2.9ab*_ln1 leaves. At 2 dpi, mock_$MgCl_2$, *Pf*0-1 EV and *Pf*0-1 *XopQ* were infiltrated into the same leaf sectors and samples were collected at 6 hpi for the biochemical fractionation assay. Two independent experiments showed similar trends.

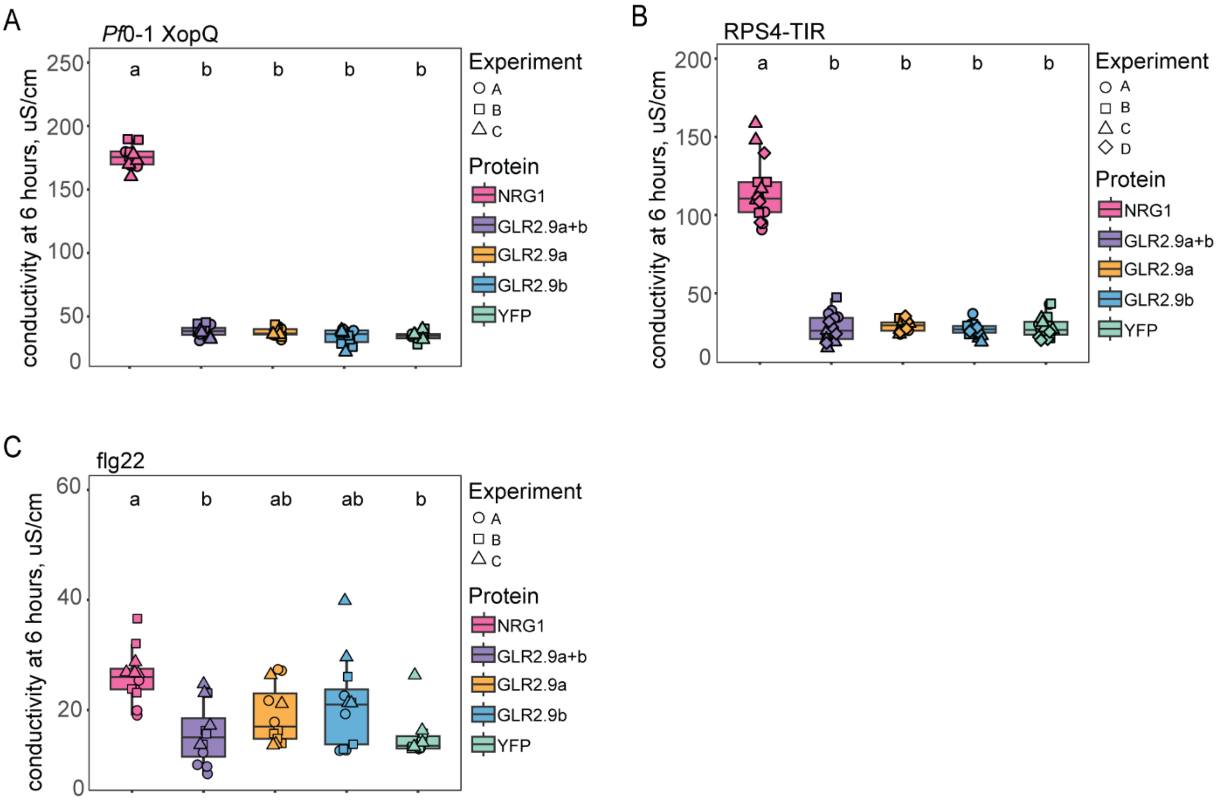

Fig. S9. Transient overexpression of GLR2.9a and/or GLR2.9b does not alleviate *nrg1-5* cell death defects in immune-triggered tissues. ***(A)*** Overexpression of GLR2.9a and/or GLR2.9b does not restore Roq1-triggered cell death in *nrg1-5*. Agrobacteria expressing *pNRG1:NRG1-4xmyc*, *p35S:gGLR2.9a-mCherry* with *p35S:gGLR2.9b-GFP* combined, *or with p35S:gGLR2.9a-mCherry*, *p35S:gGLR2.9b-GFP* or *p35S:YFP* alone were infiltrated into *nrg1-5* leaves. At 2 d post agroinfiltration, *Pf*0-1 *XopQ* was infiltrated and all samples were collected at 1 d for conductivity measurements (n = 12 from three independent experiments). ***(B)*** Overexpression of GLR2.9a and/or GLR2.9b does not restore RPS4-TIR-triggered cell death in *nrg1-5*. The same plasmids as used in *(A)* were agro-infiltrated with *RPS4-TIR* into *nrg1-5*. At 3 dpi, samples were collected for conductivity measurements (n = 16 from four independent experiments). ***(C)*** Overexpression of GLR2.9a and/or GLR2.9b does not increase cell death in *nrg1-5* in response to flg22 (1 *μM)* treatment. The same plasmids as used in *(A)* were infiltrated into *nrg1-5*. At 2 d post agroinfiltration, 1 *μM* flg22 was infiltrated and samples were collected at 1 d for conductivity measurements (n = 12 from three independent experiments). (***A***-***C***) Genotypes with different letter codes are significantly different. A one-way ANOVA followed by post hoc test was applied (α = 0.05).

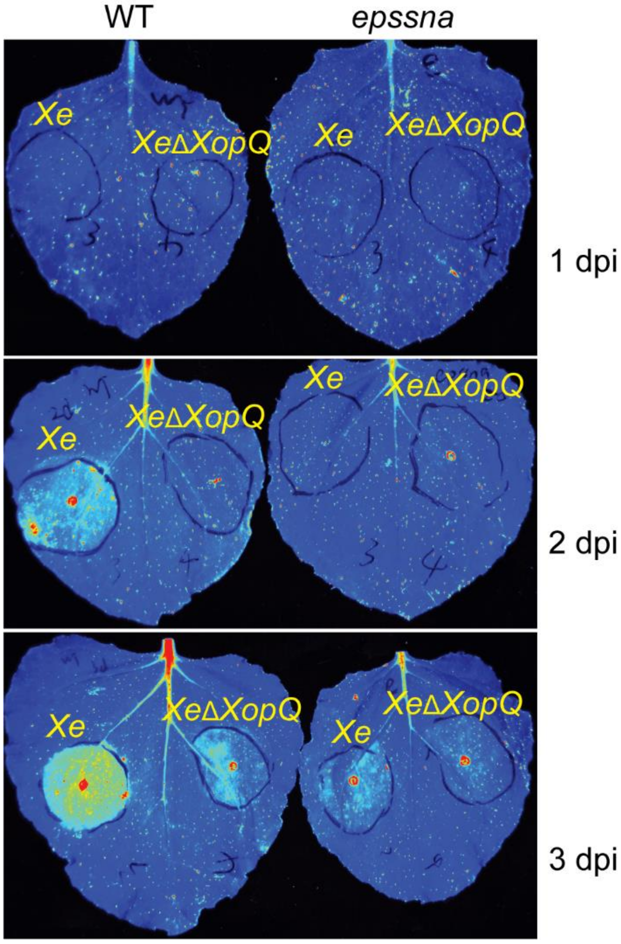

Fig. S10. *Xe* or *Xe*Δ*XopQ* induced macroscopic cell death in *Nb*. *Xe* or *Xe*Δ*XopQ* bacterial strains were infiltrated into areas of *Nb* WT and *epssna* shown by black circles. Cell death images were taken under 488 light at the indicated times post infiltration. Macroscopic cell death was first detected at 2 dpi with *Xe* in *Nb* WT.

Table S1. Oligonucleotide sequences used in this study.

Primer ID	Orientation	Sequence Information	Purpose
nJW021	5' - 3'	ttGAAGACaaAATG ACTTCTAAGCCATTCCCTAT	Golden gate cloning: Insert genomic *NbGLR2.9a* to Level 0 construct
nJW022	5' - 3'	ttGAAGACaaCGAAcc ATCTGATCTTAAATTCCCCAT	Golden gate cloning: Insert genomic *NbGLR2.9a* to Level 0 construct
nJW023	5' - 3'	ttGAAGACaaGGAG TAGAAGCAACGTCCCTGGTT	Golden gate cloning: Insert promoter region of *NbGLR2.9a* to Level 0 construct
nJW024	5' - 3'	ttGAAGACaaCATT GCTTTTTTCTACTTAGCTGA	Golden gate cloning: Insert promoter region of *NbGLR2.9a* to Level 0 construct
nJW025	5' - 3'	ttGAAGACaaAATG GGTGCAATTGTTGACATAAA	Golden gate cloning: Insert genomic *NbGLR2.9b* to Level 0 construct
nJW026	5' - 3'	ttGAAGACaaCGAAcc ATTTATGTCTTTCACACTTGGT	Golden gate cloning: Insert genomic *NbGLR2.9b* to Level 0 construct
nJW027	5' - 3'	ttGAAGACaaGGAG GGTTCCGAAACCGTATGGTAG	Golden gate cloning: Insert promoter region of *NbGLR2.9b* to Level 0 construct
nJW028	5' - 3'	ttGAAGACaaCATT TTCTATGGTTTCATTTGCAAC	Golden gate cloning: Insert promoter region of *NbGLR2.9b* to Level 0 construct
nJW033	5' - 3'	ttGAAGACaaATCG CCAAAGTGGCACTTGGT	Site-directed mutagenesis of BpiI recognition site in genomic *NbGLR2.9a*
nJW034	5' - 3'	ttGAAGACaaCGAT GACCCTCTCTCCTCTAC	Site-directed mutagenesis of BpiI recognition site in genomic *NbGLR2.9a*
nJW005	5' - 3'	ttGAAGACaaGGAG TCACTGGTTCCAAATTTTTTT	Golden gate cloning: Insert promoter region of *NbNRG1* to Level 0 construct
nJW006	5' - 3'	ttGAAGACaaCATT TGCTACATCCCGCAAAATTG	Golden gate cloning: Insert promoter region of *NbNRG1* to Level 0 construct
nJW007	5' - 3'	ttGAAGACaaACTC AACACAAAGGTTAAACATGTG	Site-directed mutagenesis of BpiI recognition site in promoter *NbNRG1*
nJW008	5' - 3'	ttGAAGACaaGAGT CCGAGTGTGAATTTACAATGC	Site-directed mutagenesis of BpiI recognition site in promoter *NbNRG1*
nDL709	5' - 3'	GGTCCAGTTTTTGCTATCCTACT CAAAGCCGTTCTTGCTGTAGGC ATAAAAATTGCC	*NbNRG1* mutagenesis D16A/D24A
nDL710	5' - 3'	GGCAATTTTTATGCCTACAGCAA GAACGGCTTTGAGTAGGATAGC AAAAACTGGACC	*NbNRG1* mutagenesis D16A/D24A
nDL707	5' - 3'	TGGTTCTTTCTGCTCCTGCTGGC TGTGCAGCGACTACTTTGGCTG CAATGCTTT	*NbNRG1* mutagenesis P-loop
nDL708	5' - 3'	AAAGCATTGCAGCCAAAGTAGT CGCTGCACAGCCAGCAGGAGCA GAAAGAACCA	*NbNRG1* mutagenesis P-loop
NbPR5-101F	5' - 3'	cgtgaagatgtgggttgatg	Forward qPCR primer for *NbPR5*
NbPR5-101R	5' - 3'	tacgccacaccacctgagta	Reverse qPCR primer for *NbPR5*
nJW063	5' - 3'	AGCAACTGCAAATGAAACCA	Forward qPCR primer for *NbGLR2.9a*
nJW064	5' - 3'	TGGAACTTGATGCCTCTTCC	Reverse qPCR primer for *NbGLR2.9a*
nJW065	5' - 3'	TGGGTGCAATTGTTGACATA	Forward qPCR primer for *NbGLR2.9b*
nJW066	5' - 3'	TGCCTTTTCCCAATATCAGC	Reverse qPCR primer for *NbGLR2.9b*
*Nb*Actin-80F	5' - 3'	gtcctggattctggtgatgg	Forward qPCR primer for *NbACT2*

*Nb*Actin-80R	5' - 3'	agacggaggatagcatgtgg	Reverse qPCR primer for *NbACT2*
oXS179	5' - 3'	GTTGACATAAATTCCCGTAT	gRNA1 for targeting *NbGLR2.9a* and *NbGLR2.9b*
oXS181	5' - 3'	GTCCTGAATTGCATCCTTGC	gRNA2 for targeting *NbGLR2.9a* and *NbGLR2.9b*
nJW309	5' - 3'	attgaaagtaaagctgcttg	gRNA1 for targeting *NbNRG1*
nJW310	5' - 3'	tggctgtggaaagactactt	gRNA2 for targeting *NbNRG1*
nJW311	5' - 3'	GTCGACGATGAGCTGCTCGG	gRNA1 for targeting *NbADR1*
nJW312	5' - 3'	ATAAAGATTGGAGTTGACGA	gRNA2 for targeting *NbADR1*
nJW313	5' - 3'	TTAATGGGTAAAGAAGATAG	gRNA3 for targeting *NbADR1*
nJW314	5' - 3'	TTCTCTGATTTACATCCCCA	gRNA4 for targeting *NbADR1*

Dataset S1 (separate file). Lists of PTI- and ETI-specific DEG in Nb WT, nrg1-5, epss and epssna.

Dataset S2 (separate file). Lists of ETI-specific Go terms in Nb WT.
